# Lipid Nanoparticle Library Screen Reveals Lymphatic Endothelial Cell-Targeting Lipid Nanoparticle for Delivering Vascular Endothelial Growth Factor C mRNA after Lymphatic Injury

**DOI:** 10.1021/acsnano.5c12080

**Published:** 2025-11-21

**Authors:** Eleftheria Michalaki, Kiyoung Jeong, Rachel Chin, Zhiming Qi, Lauren Liebman, Yarelis González-Vargas, Elisa Schrader Echeverri, Kalina Paunovska, Hiromi Muramatsu, Norbert Pardi, Beth Jiron Tamburini, Zoltan Jakus, James E. Dahlman, J. Brandon Dixon

**Affiliations:** † George W. Woodruff School of Mechanical Engineering, 1372 Georgia Institute of Technology, Atlanta, Georgia 30332, United States; ‡ Wallace H. Coulter Department of Biomedical Engineering, Georgia Institute of Technology and Emory University, Atlanta, Georgia 30332, United States; § Department of Biology, Georgia Institute of Technology, Atlanta, Georgia 30332, United States; ∥ Parker H. Petit Institute for Bioengineering and Bioscience, Georgia Institute of Technology, Atlanta, Georgia 30332, United States; ⊥ Department of Microbiology, Perelman School of Medicine, 6572 University of Pennsylvania, Philadelphia, Pennsylvania 19104, United States; # 129263 University of Colorado School of Medicine, Department of Medicine, Aurora, Colorado 80045, United States; ∇ 316970 Semmelweis University School of Medicine, Department of Physiology, Budapest 1085, Hungary

**Keywords:** lipid nanoparticle, mRNA, lymphatic endothelial cell, lymphedema, VEGFC, lymphangiogenesis

## Abstract

Dysfunction of the lymphatic system following injury, disease, or cancer treatment can lead to lymphedema, a debilitating condition with no cure. Advances in targeted therapy have shown promise for treating diseases where conventional therapies, including broad-spectrum chemotherapy, surgical interventions, and palliative care, have often failed to achieve durable responses. As lymphatic vessels have recently emerged as a therapeutic target, nucleic acid delivery via nanoparticles to lymphatic endothelial cells (LECs) has been studied utilizing lymphatic drainage; however, there are no approaches to modulate the chemical compositions of lipid nanoparticles (LNPs) to optimize LEC specificity. To identify an LNP that effectively delivers mRNA to LECs after intradermal (ID) injection, we screened a library of 150 LNPs in total spread out over 3 screening rounds. The 150 formulations had a variety of chemical compositions and were assessed for both self-assembly and delivery *in vivo* to LECs. We identified and validated several LNP formulations optimized for high LEC uptake when administered ID and compared their efficacy for delivery of functional mRNA with that of free mRNA and mRNA delivered with a commercially available MC3-based LNP (Onpattro). The lead LEC-LNP was then loaded with VEGFC mRNA to test the therapeutic advantage of this LEC enrichment (namely, LNP7) for treating a mouse tail lymphatic injury model. A single dose of VEGFC mRNA delivered via LNP7 resulted in enhanced LEC proliferation at the site of injury and an increase in lymphatic function up to 14 days postsurgery. Our results suggest a therapeutic potential of VEGFC mRNA using lymphatic-enriched LNP delivery for alleviating lymphatic dysfunction observed during lymphatic injury and could provide a promising approach for targeted and transient lymphangiogenic therapy.

## Graphical abstract

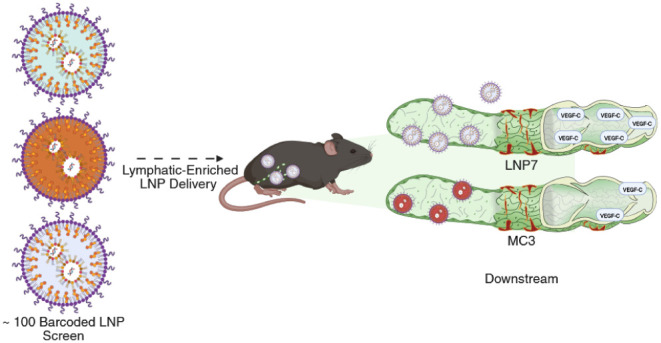

## Introduction

The lymphatic system helps maintain fluid balance in the body by draining excess interstitial fluid from tissues and depositing it into the bloodstream.^1^ Lymph flow is driven by both active lymphatic wall pumping and transient drops in fluid pressure due to the contraction of surrounding tissue to promote the uptake of fluid from the interstitium to the initial lymphatic capillaries and through the lymphatic network.^2–4^ Specialized lymphatic muscle lines the collecting lymphatic vessels (LVs) downstream of the initial LVs.^5^ Sections of LVs, where smooth muscle is present (lymphangions), perform spontaneous muscle contractions. The presence of lymphatic valves,^4,6^ in conjunction with the active contraction cycle of individual lymphangions, makes the intrinsic pump highly effective for promoting unidirectional flow.^2^

Damaged LVs become leaky, and their efficiency for pumping fluid is reduced, leading to the excessive accumulation of fluid, macromolecules, and leukocytes.^7^ This accumulation, caused by lymphatic impairment, is associated with diseases including edema, fibrosis, obesity, cardiovascular disease, and neurological disorders.^7–10^ Furthermore, dysfunction of the lymphatic system can result in poor immune function and lead to inflammation,^11^ autoimmune diseases,^12^ impaired wound healing,^8^ and even tumor progression.^13^ Therefore, the lymphatic system is not only important for fluid transport but also in modulating immune function.^12^ As an example, lymphatic endothelial cells (LECs), comprising lymphatic capillaries, influence dendritic cell maturation.^12,14^

Given the significant impact of the lymphatic system and LECs on multiple pathophysiological conditions, several studies have explored various treatments to address lymphatic dysfunction. Clinically, the amelioration of lymphatic dysfunction in disease conditions focuses on palliative^15–18^ or surgical treatments, such as vascularized lymph node transplantation.^19–24^ However, patients receive these treatments for extended periods with high costs, and their efficacy remains limited, indicating the need for a pharmacotherapy strategy to achieve efficient therapy *in vivo*.^1^

Reduction of inflammation has been used for the treatment of lymphatic dysfunction.^20^ For instance, ketoprofen, a nonsteroidal anti-inflammatory drug (NSAID), has been shown to not only inhibit the inflammatory pathways of both cyclooxygenase (COX) and 5-lipoxygenase (5-LO) but also to promote lymphatic repair.^25^ However, NSAIDs carry the risk of heart attack or stroke.^26^ In addition to the inhibition of inflammatory pathways, growth factors, such as fibroblast growth factor 2 (FGF2), hepatocyte growth factor (HGF), retinoic acid (RA), and vascular endothelial growth factor C (VEGFC), have been studied in preclinical models as potential agents to induce lymphangiogenesis and improve lymphatic function. FGF2 and RA, however, induce lymphangiogenesis indirectly by upregulating VEGFC expression.^27^ VEGFC induces lymphangiogenesis by binding to vascular endothelial growth factor receptor 3 (VEGFR3).^28,29^ VEGFC-mediated lymphangiogenesis expands the lymphatic network, limits inflammation,^14^ and improves lymphatic function.^20^

Nanoparticles (NPs) have been recently utilized as robust delivery agents by encapsulating or attaching therapeutic drugs and distributing them to target tissues.^30–32^ To deliver VEGFC protein to LECs, VEGFC protein-loaded biodegradable NPs have been recently studied with a variety of approaches, including poly lactic-*co*-glycolic acid nanosphere,^33^ gelatin hydrogel,^34^ and mesenchymal stem cells (MSCs).^35,36^ While these protein-based strategies have shown promising preclinical outcomes, their clinical translation remains limited. Protein therapies are hindered by a short plasma half-life, poor stability in physiological conditions, aggregation, and the potential to elicit immune responses.^37–41^ These limitations necessitate repeated dosing and raise safety concerns, thereby reducing the therapeutic durability and scalability of protein delivery in chronic conditions such as lymphedema.

Advances in genomics have led to the development of targeted gene therapies.^42^ To date, siRNA therapies have shown the most promise in patients with infectious and cardiometabolic diseases,^43,44^ while adenovirus-based platforms only recently were utilized for patients with lymphatic dysfunction—a clinical trial based on an adenovirus-based VEGFC delivery platform (Lymfactin) entered Phase II (NCT03658967).^45,46^ Although Lymfactin did not exhibit adverse effects such as AAV-related immunogenicity or toxicity during Phase 1, adverse effects were observed in Phase 2—ranging from minor symptoms like cold, fever, gastroenteritis, and elevated liver enzymes to serious events such as erysipelas infection and hematoma. Improvement in excess arm volume was seen only after 12 months in conjunction with surgery. Furthermore, the clinical study involving AAV vectors yielded inconclusive results, leading to the discontinuation of the drug’s development.^45–47^ Lastly, these approaches do not allow for the temporary upregulation of protein production that can be achieved with mRNA. Amid these challenges of adenovirus-based approaches, mRNA-based platforms offer a simpler and less expensive alternative with higher therapeutic efficacy than proteins due to their continuous translation and higher transfection efficiency. Moreover, they have lower toxicity than DNA-based or adenovirus- and adeno-associated virus-based platforms, as they do not require entry into the nucleus to be functional^. *^37,39,48^*^

To achieve targeted RNA delivery to LECs, lipid nanoparticles (LNPs), an RNA vehicle, are designed to protect nucleic acids and transport them to the target cells while minimizing off-target effects.^49^ Although the soluble nature of VEGFC and immune cell involvement in VEGFC delivery might seem to dilute the importance of LEC targeting,^50^ if LECs could enhance the secretion of their own growth factor, this could guide regeneration in the context of an injury, as this VEGF-C would leak out of the injured lymphatic. It has been previously shown that there is a synergy between interstitial flow and growth factors to drive not only LEC organization but also the creation of gradients relative to the cell that enhances morphogenesis, a feature that could perhaps be leveraged with mRNA delivery of VEGF-C to LECs.^51–53^

In conjunction with rapid NP synthesis, high-throughput *in vivo* NP screening methods allow scientists to track many LNPs simultaneously, allowing one to identify organ-specific LNPs in a more efficient manner. Specifically, high-throughput DNA barcoding systems have been developed that allow analysis of >100 LNPs *in vivo*.^54–58^ Notably, simultaneous administration of many LNP formulations overcomes challenges associated with expensive *in vivo* screening and existing physical (e.g., brain accessibility, LNP disassembly) and physiological (e.g., undesired LNP binding to serum proteins) barriers.^55^ Several barcoding assays have been reported, a subset of which quantify functional mRNA delivery (i.e., delivered mRNA turning into protein) mediated by many LNPs at once. One such assay is called Species Agnostic Nanoparticle Delivery Screening (SANDS),^56^ which measures the functional delivery of mRNA encoding an anchored VHH antibody (aVHH)—single-domain camelid antibodies that neutralize respiratory syncytial virus (RSV)—linked to the decay-accelerating factor (DAF)-GPI membrane anchor sequence to retain the antibodies on the plasma membrane of transfected cells.^57^ Given the implication of the lymphatic system in various disease conditions and the challenges of lymphatic-specific targeting, the multiplexity of DNA barcoding technology combined with the versatility of LNPs may be a means to accelerate the development of lymphatic-specific genetic therapies.

Here, we used SANDS to study how 150 different LNP formulations delivered mRNA and identified several LNPs *in vivo* that deliver functional mRNA to LECs. We then used the leading LNP (named LNP7) to deliver VEGFC mRNA to LECs, thereby improving lymphatic regeneration and function following lymphatic injury.

## Results/Discussion

### Generation of LNP Library for LEC Delivery at LN

To guide the library design, we selected lipid components based on prior evidence that ionizable lipid tail length and stereochemistry influence endosomal escape, cholesterol polarity affects particle stability and cellular uptake, PEG chain length modulates circulation and lymphatic drainage, and helper lipid geometry (cylindrical vs cone-shaped) impacts membrane fusion. These considerations formed the basis for systematically varying the lipid structures in the library. Using this initial library of components, we first used SANDS to identify an LEC-targeting LNP. Over three experiments, we screened a library of 150 chemically distinct LNPs by varying LNP ionizable lipid, cholesterol, alkyl-tailed PEG, and helper lipids (e.g., DOPE, DSPC) (Figures 1 and S1). We selected specific lipid components based on their availability and physicochemical properties, including lipid type and charge. For ionizable lipids, we used multitail structures with varying tail lengths (C10, C12, and C13) and different stereoisomers, as well as cKK-E12.^59^ For cholesterol, we investigated the impact of cholesterol polarity by comparing standard cholesterol with 20α-hydroxycholesterol. In the case of PEG-lipids, we compared different chain lengths using C14PEG2K and C18PEG2K. As helper lipids, we selected both neutral cylindrical-shaped DSPC and neutral cone-shaped DOPE,^60–62^ along with 18:1 cap-PE, to assess their shape or the presence of the cap group. Lastly, we included DOTAP and DDAP to evaluate the influence of positively charged helper lipids. Each chemically distinct LNP was loaded with an mRNA encoding aVHH and a unique DNA barcode. After synthesis, each LNP formulation was evaluated *in vitro* for a series of criteria outlined in detail in the methods. Ninety-nine of these unique LNP formulations passed this stage and were selected for *in vivo* delivery. At each screening, LNPs were intradermally injected in each paw of 5 mice total (3 mice with LNPs for screening and 2 mice with saline for control). Between 12 and 16 h after LNP injection, the downstream LNs were collected and digested before using fluorescence-activated cell sorting (FACS) to sort LECs with high aVHH expression (i.e., cells in which aVHH mRNA was functionally delivered). Lymph nodes were chosen for screening of LEC targeting instead of collecting lymphatic vessels due to the substantially larger number of LECs within the subcapsular sinus of the lymph node compared to the afferent lymphatics. Sorted cells were pooled across all mice and all lymph nodes, then sequenced to identify the DNA barcodes present within the cells, thereby identifying LNPs colocalized with cells in which functional delivery occurred. Screens 1 and 3 were sequenced to obtain barcode reads, whereas Screen 2 was not sequenced due to the very low number of aVHH^+^ LECs recovered (Figure S2). Unlike Screens 1 and 3, the LNPs in Screen 2 yielded very few aVHH^+^ LECs, preventing sequencing. This result was not due to technical failure but reflected the lipid compositions included, particularly DOTMA and C18PEG2K, which appear to impair LEC delivery. This finding aligns with prior evidence that shorter acyl chains are more favorable for lymphatic endothelial uptake.^63^

**Figure 1:**
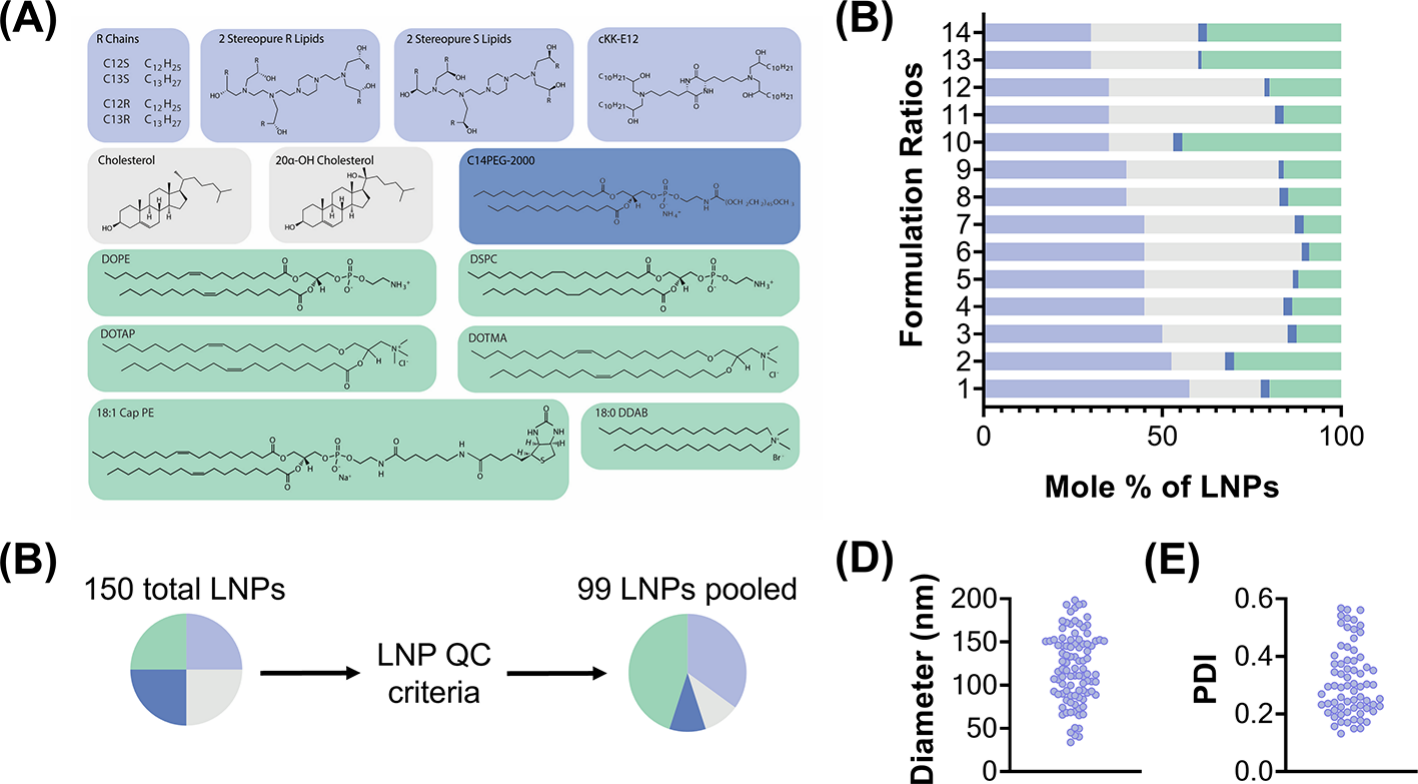
Screening different LNP formulations with SANDS. (A) Main lipid components of the LNP libraries tested. (B) Each of the compounds was formulated by using 14 molar ratios. (C) Of the 150 LNPs that were formulated, 99 passed the quality control (QC) criteria with a diameter less than 200 nm as well as a stable autocorrelation curve. (D) Hydrodynamic diameters and (E) polydispersity indexes (PDI) of all administered LNPs; the diameter of the LNP pooled control is within the range of the LNPs composing the pool.

### Identification of Lead LEC-LNP Candidates

After identifying LNPs capable of delivering functional aVHH mRNA to lymph node LECs, we selected six lead candidates (LNP1, LNP2, LNP3, LNP4, LNP7, and LNP11) based on the highest total lymph node LEC delivery values measured by SANDS among those formulations that passed QC criteria (hydrodynamic diameter <200 nm and a stable autocorrelation curve) (Figures 2A, S3 and Tables S1 and S2). We then confirmed the LEC delivery of the LNPs individually by intradermally injecting them into the paws of mice and quantifying the aVHH^+^ LECs in the axillary lymph node (ALN), brachial lymph node (BLN), and popliteal lymph node (PLN) using flow cytometry. We found that LNP1, LNP2, and LNP7 led to consistently high percentages of aVHH^+^ LECs in all examined LNs (ALN: LNP1 45%, LNP2 47%, LNP7 28% (Figure 2B); BLN: LNP1 44%, LNP2 34%, LNP7 32% (Figure 2C); PLN: LNP1 39%, LNP2 42%, LNP7 53% (Figure 2D)). Among these, LNP7 yielded the highest uptake by LECs in the PLN. The PLN is the primary draining lymph node of the hindlimb and is a well-established site for evaluating lymphatic targeting and function, as numerous murine and rat lymphedema models rely on popliteal lymph node dissection to induce sustained edema and quantify therapeutic effects.^64–66^ Accordingly, the high delivery to the PLN led us to prioritize LNP7 for subsequent in vivo validation.

**Figure 2:**
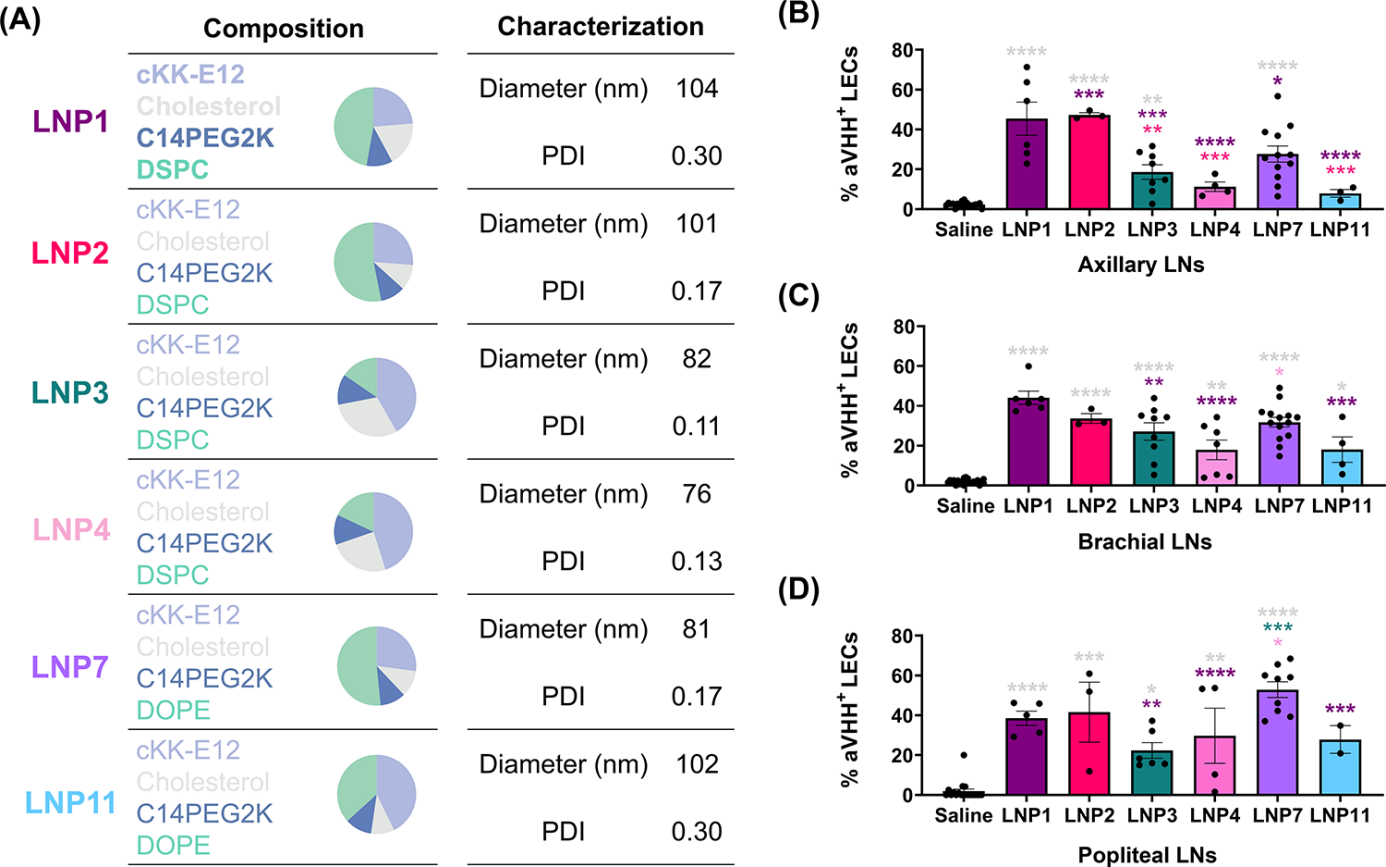
Validation of the lead LEC-LNP candidates. (A) Formulation compounds, composition, hydrodynamic diameter (nm), and PDI of lead LEC-LNPs. (B–D) Percentage of aVHH^+^ LECs (after gating for Live/Dead, CD31^+^/PDPN^+^) from (B) ALN, (C) BLN, and (D) PLN after administration with saline (gray), LNP1 (dark purple), LNP2 (magenta), LNP3 (green), LNP4 (pink), LNP7 (purple), or LNP11 (blue). Each data point corresponds to an independent experiment (ALN: *N*_Saline_ = 20, *N*_LNP1_ = 6, *N*_LNP2_ = 3, *N*_LNP3_ = 8, *N*_LNP4_ = 4, *N*_LNP7_ = 13, and *N*_LNP11_ = 3; BLN: *N*_Saline_ = 22, *N*_LNP1_ = 6, *N*_LNP2_ = 3, *N*_LNP3_ = 9, *N*_LNP4_ = 8, *N*_LNP7_ = 14, and *N*_LNP11_ = 4; PLN: *N*_Saline_ = 18, *N*_LNP1_ = 5, *N*_LNP2_ = 3, *N*_LNP3_ = 6, *N*_LNP4_ = 4, *N*_LNP7_ = 9, and *N*_LNP11_ = 2), and error bars represent the corresponding standard error of the mean (SEM). Color-coordinated asterisks above plots indicate a pairwise comparison for significance using a one-way ANOVA and robust regression and outlier removal (ROUT) method to identify and remove outliers, followed by a post hoc test to correct for multiple comparisons with *p* < 0.05 (*), *p* < 0.01 (**), *p* < 0.001 (***), and *p* < 0.0001 (****).

To identify the relationship between LNP parameters and LEC delivery, we compared the diameter, zeta potential, and encapsulation efficiency between the leading candidates and the non-LEC-targeting LNPs that passed QC at screening: LNP13, 21, 25, and 27 (Figure S4a). Subsequently, to determine the correlation between LNP parameters and LEC delivery, linear regression was performed to identify if there was any correlation between the measured parameters and LEC delivery. None of the LNP parameters showed a significant linear relationship to LEC delivery (Figure S4).

### Data-Driven Analysis of DNA-Barcoded LNP Library Showed an LNP Composition-Lymphatic Specificity Relationship

To leverage the SANDS library to better understand what LNP properties led to LEC delivery, we applied machine learning models to identify features of LNPs that influence lymphatic tissue specificity. Input features included lipid types (binary: 0 (not included) or 1 (included)) and lipid composition (continuous), and the output target is the total LN LEC delivery value from the SANDS library. In addition to linear regression, we selected tree-based models—random forest and XGBoost (XGB)—to account for potential nonlinear relationships between lipid compositions and lipid interactions, as well as constraints imposed among features. The processed data were split into training and testing sets to evaluate model performance based on the mean squared error (MSE) and the coefficient of determination (*R* ^2^) (Figure 3A).

**Figure 3:**
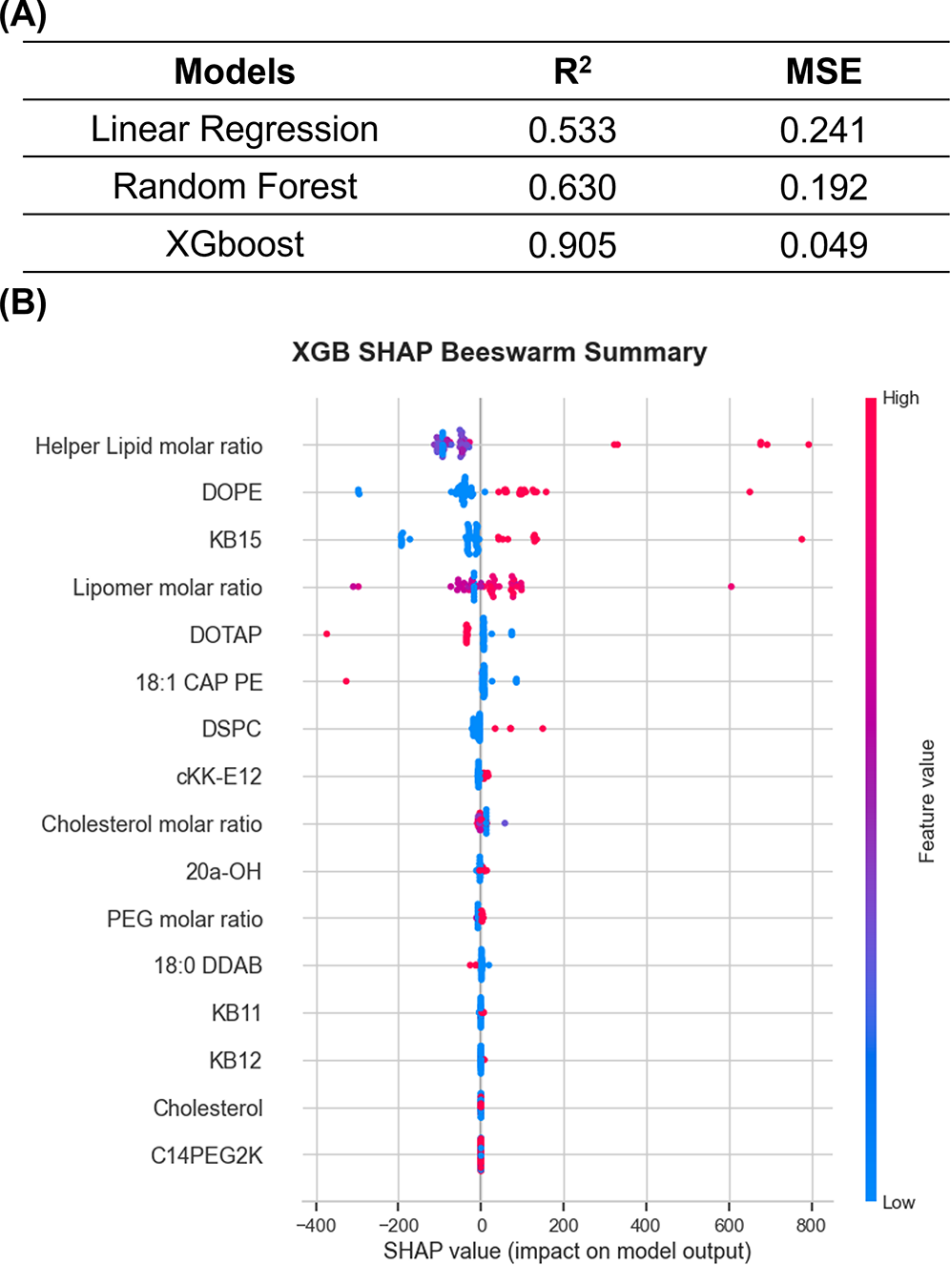
Model performance and SHAP beeswarm summary plot of XGBoost (XGB) to identify the features influencing lymphatic specificity. (A) Model performance was evaluated by mean squared error (MSE) and coefficient of determination (*R* ^2^). (B) SHAP beeswarm summary plot from the XGB model. Features are ranked by the absolute mean of their Shapley values, reflecting their relative importance in predicting LEC delivery. Positive Shapley values indicate that higher feature values increase the predicted LNP delivery, whereas negative Shapley values suggest a decrease in the prediction.

To assess the contribution and relevance of each feature, we extracted coefficients from the linear regression model and feature importance scores from the tree-based models (Figures 3B and S5). Based on performance metrics, XGB was chosen for subsequent analysis, as it achieved higher *R* ^2^ scores and lower MSE compared to the other models (Figure 3A). Local attribution plots were generated for the XGB model to visualize feature-level contributions (Figure S5c).

From the Shapley additive explanation (SHAP) analysis, the importance of features was listed based on the absolute mean of their Shapley values, reflecting their relative importance in predicting total LN LEC delivery. The helper lipid molar ratio and neutral helper lipids—particularly DOPE—were ranked as the first and second highest absolute mean of their Shapley values, identified as the primary determinants driving LEC uptake in this screening. Considering that no lymphatic-specific LNP candidates were formulated with DOTAP or DDAP, and that lower feature values (in this case, 0 or not included in that LNP) were associated with positive SHAP values, cationic helper lipids are not expected to be favorable options for lymphatic-targeting LNPs, which is also supported by the low LEC cell number in screen 2, which included the cationic helper lipid DOTMA (Figures S1 and S2). Notably, neutral 18:1 cap PE also showed a negative correlation with LEC delivery, suggesting that the presence of a cap at the headgroup may reduce delivery efficiency.

Although DOPE is present in both high- and low-performance formulations, SHAP analysis showed that its inclusion was positively correlated with lymphatic endothelial delivery. The variability observed among DOPE-containing LNPs likely reflects the influence of additional features, such as helper lipid ratios and the absence of cationic helper lipids. Thus, while DOPE is generally favorable, delivery efficiency is dependent on the overall lipid composition.

As ApoE-mediated uptake has previously been identified as a promising mechanism for lymphatic delivery of LNPs,^67^ the PEG molar ratio also plays a significant role and warrants further investigation. However, the lymphatic specificity observed in the leading LNP candidates does not appear to be predominantly driven by the PEG molar ratio. Prior research indicated that within a specific LNP formulation, 6% PEG yields the highest LEC delivery, whereas in this study, the PEG content ranged from 1 to 2.5% and ranked lower in feature importance. These findings suggest that LNP lipophilicity or LEC affinity may instead be modulated by other components, particularly helper lipids, in the formulations tested.

### LNP7 Provides Superior Functional mRNA Delivery to LEC in Both the Draining Lymphatic Vessel and the Draining Lymph Node

Next, we sought to demonstrate the superiority of LNP7, which led to the highest LEC uptake in the PLN, in delivering mRNA cargo to LECs *in vivo* in both the LEC of the afferent collecting lymphatic vessel and the draining lymph node. We compared LNP7 delivery of aVHH mRNA to LECs of the PLN to administration of saline, free mRNA (aVHH mRNA), and MC3-based LNPs (MC3) loaded with aVHH mRNA, an FDA-approved hepatocyte-targeting LNP formulation in Onpattro^68,69^ (Figure 4). In this study, we injected only one hindlimb, which allowed us to use the lymph nodes that drain the remaining limbs as controls for off-target delivery. After 12–16 h of administration, we isolated the PLN draining the injection site (PLN Injected) from the injection site and the ALN, the BLN, and the contralateral PLN (PLN Contralateral) from the noninjected sites and quantified the percentage of LEC that express aVHH using flow cytometry (Figure 4, Tables S3 and S4). LNP7 led to the highest mRNA delivery to LECs in the PLN Injected (saline 2%, free aVHH 2%, MC3 5%, LNP7 31%). There was no significant difference in functional mRNA delivery when comparing LNP7 and MC3 in nodes that drain the noninjected sites (ALN, BLN, and PLN Contralateral), demonstrating that the enhanced LEC delivery of LNP7 is specific to LECs of the lymph node that directly drains the injection site (Figure S6). In an additional study, we dissected the afferent popliteal LVs (PLV) and, upon quantification with FACS, found that LNP7 consistently led to the highest cargo delivery to LECs in collecting LVs (saline 1%, free aVHH 2%, MC3 10%, LNP7 37%) (Figure 4A and Table S3). In addition, we investigated the delivery of LNPs in other endothelial and immune cell populations that reside in the draining and nondraining LNs using a 12-channel flow cytometry with a gating strategy detailed in the provided Supporting Information. aVHH expression remained low (below ∼15%) for all examined cell populations except for dendritic cells (cDC2 and cDC1), where we saw a significant uptake of LNP7 in the injected PLN (∼42% and ∼28%, respectively (Figures 4B and S6). aVHH expression also remained low for all cell populations in the nondraining lymph nodes (Figure S6). We then evaluated LNP7 on LEC viability *in vitro* and found no evidence of overt toxicity at all of the doses tested (Figure S7).

**Figure 4:**
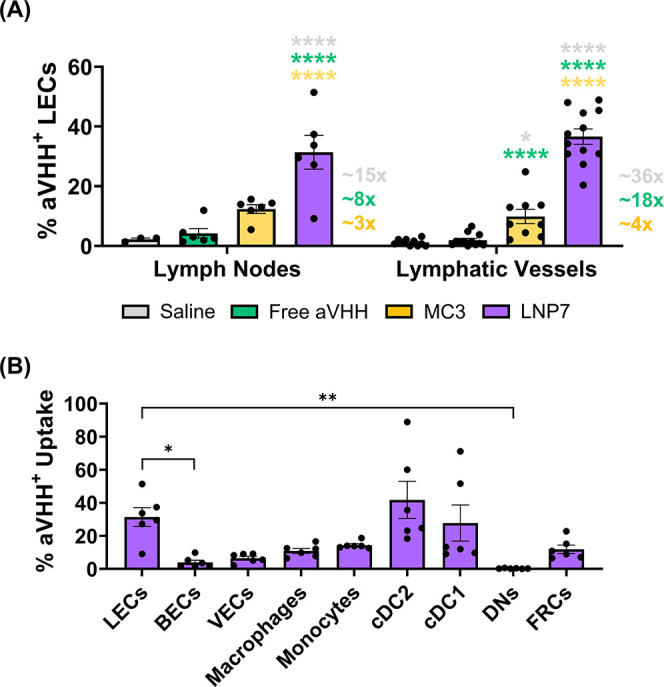
LNP7 provides superior delivery of mRNA to LECs and DCs within the draining lymph node and to LECs within the collecting lymphatic vessels. (A) Percentage of aVHH^+^ LECs from PLN and PLV after delivery with saline (gray), free aVHH (green), MC3 (gold), and LNP7 (purple). (B) Percentage of aVHH^+^ cells upon delivery with LNP7 by different cell types within the draining lymph node, namely, LECs, blood endothelial cells (BECs), vascular endothelial cells (VECs), macrophages, monocytes, dendritic cells (cDC2, cDC1), double-negative cells (DNs), and fibroblastic reticular cells (FRCs). Each data point corresponds to an independent experiment (Lymph Nodes: *N*_Saline_ = 3, *N*_Free aVHH_ = 6, *N*_MC3_ = 6, and *N*_LNP7_ = 6; Lymphatic Vessels: *N*_Saline_ = 10, *N*_Free aVHH_ = 12, *N*_MC3_ = 9, and *N*_LNP7_ = 12), and error bars represent the SEM. (A) Color-coordinated asterisks above plots indicate a pairwise comparison for significance using a two-way ANOVA with Tukey’s multiple comparisons test with *p* < 0.05 (*) and *p* < 0.0001 (****). (B) Solid lines above plots indicate a pairwise comparison for significance using a one-way ANOVA with Tukey’s multiple comparisons test with *p* < 0.05 (*) and *p* < 0.01 (**). Identification of the respective cell populations (after Live/Dead gating) was as follows: LECs: CD45^–^/CD31^+^/PDPN^+^, VECs: CD45^–^/CD31^+^/PDPN^–^/CD54^+^, BECs: CD45^–^/CD31^+^/PDPN^–^/CD309^+^, FRCs: CD45^–^/CD31^–^/PDPN^+^, DNs: CD45^–^/CD31^–^/PDPN^–^, Monocytes: CD45^+^/CD11b^+^/CD64^+^/F4–80^–^, Macrophages: CD45^+^/CD11b^+^/CD64^–^/F4–80^+^, cDC2: CD45^+^/CD11c^+^/MHCII^+^/CD11b^+^, and cDC1: CD45^+^/CD11c^+^/MHCII^+^/CD11b^–^.

### *In Vivo* Mouse Biodistribution Studies Revealed No Significant Systemic Spillover Following Intradermal Injection of LNPs

To elucidate the time-dependent biodistribution of the LNPs, mice were intradermally administered 1 μg of miRNA conjugated with Cy3 into the right paw and sacrificed at 1, 4, and 24 h postinjection. Isolated lymph nodes (popliteal lymph node at the injection site (PLNI), popliteal lymph node at the contralateral site (PLNC), axillary lymph node at the injection site (ALN), and brachial lymph node at the injection site (BLN)), spleens, and livers were imaged using the *In Vivo* Imaging System (IVIS) to quantify Cy3 fluorescence. Representative images (Figure 5A) and their quantification of the average radiant efficiency (Figure 5B) showed that Cy3 uptake by the PLN injection site (PLNI) peaked 4 h after injection for all groups, and LNP7 showed the highest uptake at the PLNI without showing systemic spillover to the spleen and liver compared to the PBS group. Interestingly, the MC3-based LNP, known to be a liver-targeting LNP when delivered intravascularly, showed no difference in the liver uptake, implying that the administration route is a more significant factor than chemistries or lipid ratios. LNP7 showed either overall higher uptake or no difference when compared to MC3 at the other LNs that do not serve as the primary lymph node draining the injection site (PLN at the contralateral side (PLNC), BLN, and ALN) (Figure 5B).

**Figure 5:**
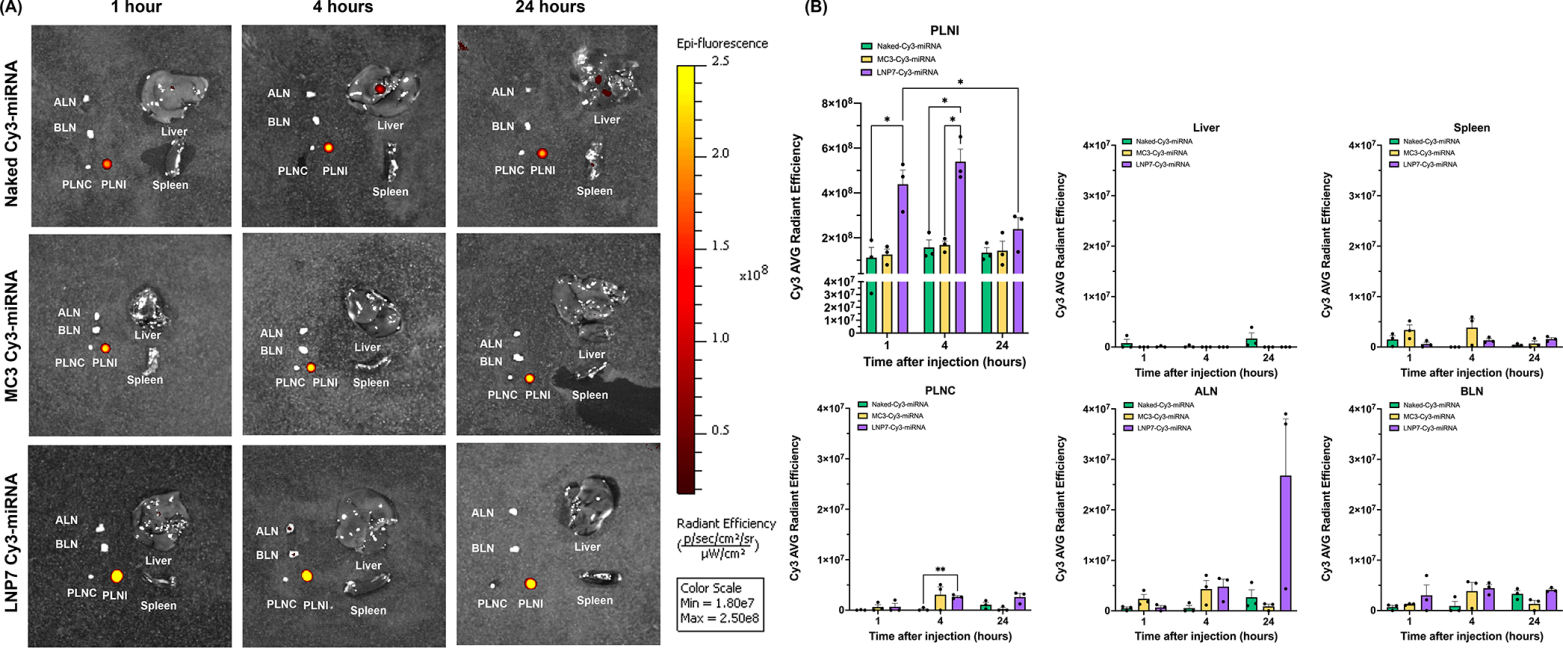
LEC-LNP encapsulation of Cy3-conjugated miRNA showed a higher uptake in the PLN at the injection site (PLNI) without significant systemic spillover. LNP7 uptake by PLNI peaked at 4 h and reduced 24 h after injection. The biodistribution of LNPs loaded with Cy3-miRNA and naked Cy3-miRNA was measured after 1, 4, and 24 h of intradermal administration. Cy3 average radiant efficiency was measured at the popliteal lymph nodes contralateral/injection site (PLNC/PLNI), the brachial lymph node at the injection site (BLN), and the axillary lymph node at the injection site (ALN) for lymphatic distribution. Cy3 average radiant efficiency at the spleen and liver was assessed for systemic spillover. Images (A) and the quantification (B) are above. Two-way ANOVA: the Tukey test was used for multiple comparisons. * for *p* < 0.0332, ** for *p* < 0.0021, **** for *p* < 0.0001, respectively.

### LEC Targeting Has No Benefit at the Injection Site

Before proceeding to the injury model, to ensure the effect of VEGFC mRNA-LNPs on lymphatic growth and LEC proliferation, we injected 1 μg of VEGFC mRNA encapsulated in LNPs intradermally into the ear dermis. Given that the objective of these experiments was to determine if there was a functional effect of the respective LNP platform when loaded with VEGF-C mRNA, in this and subsequent VEGF-C mRNA experiments, we always included an LNP7 loaded with aVHH mRNA and no VEGF-C mRNA. The rationale for this choice was to have an LNP with similar properties to ensure the effect was not due to the LNP itself but rather the VEGF-C mRNA. Lymphatic growth (Figure 6A–C) and LEC proliferation as measured by EdU (Figure 6D) were assessed 14 days after injection and compared with LNP7-aVHH mRNA. Harvested ear samples were whole-mounted, IF-stained, and imaged by confocal microscopy.

**Figure 6:**
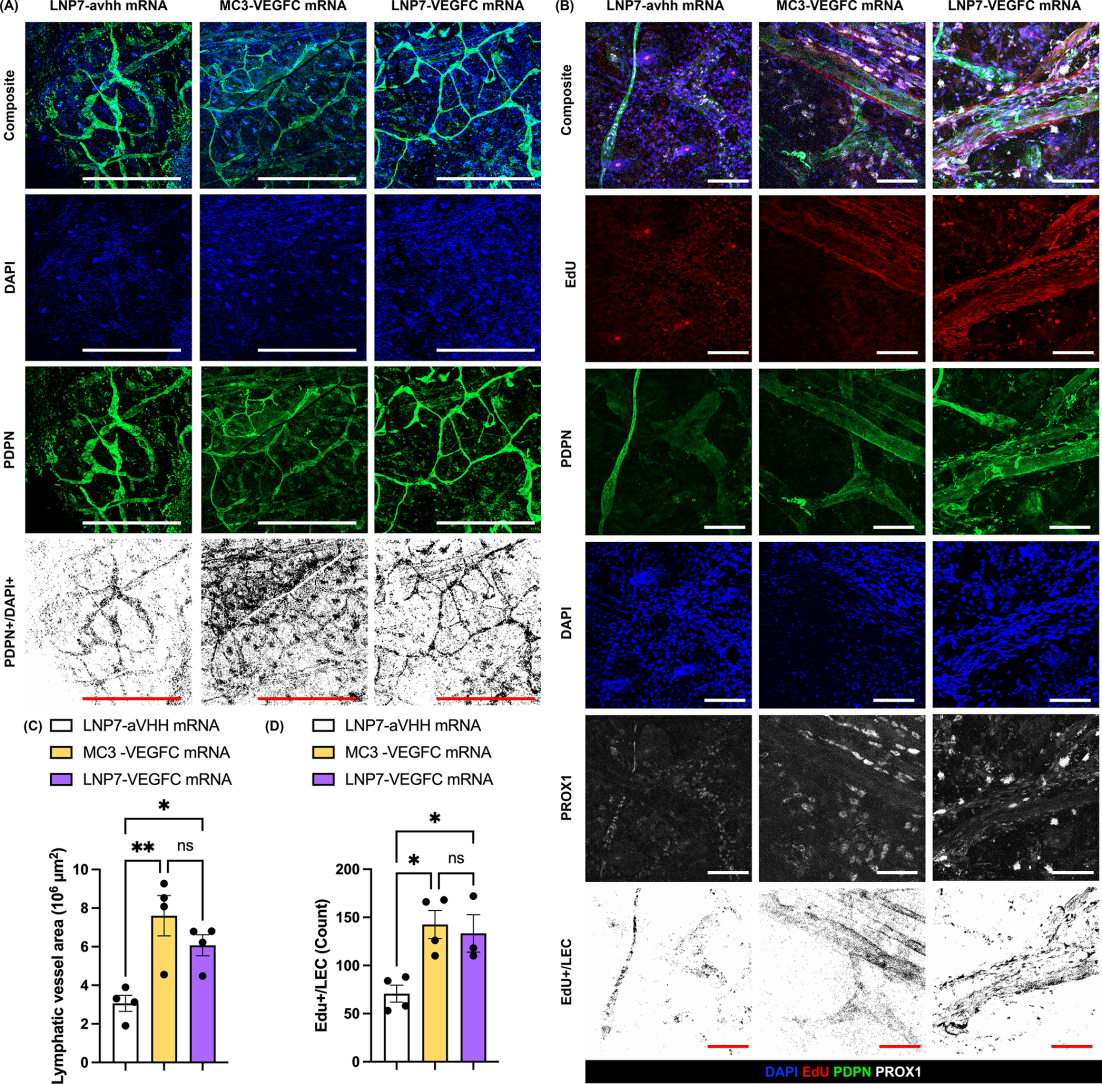
LNPs with VEGFC mRNA showed lymphatic vessel growth and cell proliferation at the injection site. (A) Assessment of lymphatic vessel growth 14 days after intradermal injection on mice ears with 1 μg of LNP7-aVHH mRNA, MC3-VEGFC mRNA, and LNP7-VEGFC mRNA. Anti-PDPN and DAPI immunofluorescence staining were imaged on whole-mounted ear samples by confocal microscopy. Representative images from each group were shown (bars, 1000 μm). (B) Assessment of lymphatic proliferation 14 days after intradermal injection on the ears of mice with 1 μg of LNP7-aVHH mRNA, MC3-VEGFC mRNA, and LNP7-VEGFC mRNA. Anti-PDPN, anti-PROX1, DAPI, and EdU immunofluorescence staining were imaged on whole-mounted ear samples by confocal microscopy. Representative images from each group were shown (bars, 100 μm). (C) The colocalized areas between PDPN and DAPI were measured. One-way ANOVA: Tukey test for multiple comparisons. * for *p* < 0.05, ** for *p* < 0.005, respectively. (D) Nuclei colocalized with EdU, PROX1, PDPN, and DAPI were counted. One-way ANOVA: Tukey test for multiple comparisons. * for *p* < 0.05.

Both MC3 and LNP7 encapsulating VEGFC mRNA (MC3-VEGFC mRNA and LNP7-VEGFC mRNA) significantly increased the PDPN^+^/DAPI^+^ LEC area compared to LNP7-aVHH mRNA. MC3-VEGFC mRNA exhibited a slightly higher PDPN^+^/DAPI^+^ area but showed no significant difference compared to that of LNP7-VEGFC mRNA.

The proliferation of LEC was also quantified based on the cell counts colocalized between PDPN, PROX1, DAPI, and EdU (Figure 6B,D). Similarly, the higher proliferation of LEC from VEGFC mRNA-encapsulating LNPs was observed compared to LNP7-aVHH mRNA, with no difference between VEGFC mRNA-LNPs.

### Lymphatic-Targeting LNP Induces Higher VEGFC Secretion in the Downstream Fluid in the Popliteal Dermal and Subcutaneous Region

To investigate the time-dependent secretion of VEGFC at both the injection site and the downstream popliteal region, 1 μg of VEGFC mRNA encapsulated in LNPs was intradermally injected into the hindlimb mouse paw. At specified time points (1, 4, 7, and 14 days postinjection), VEGFC protein levels in the subcutaneous and dermal layers of the popliteal region and in the paw skin at the injection site were quantified by ELISA (Figure 7).

**Figure 7:**
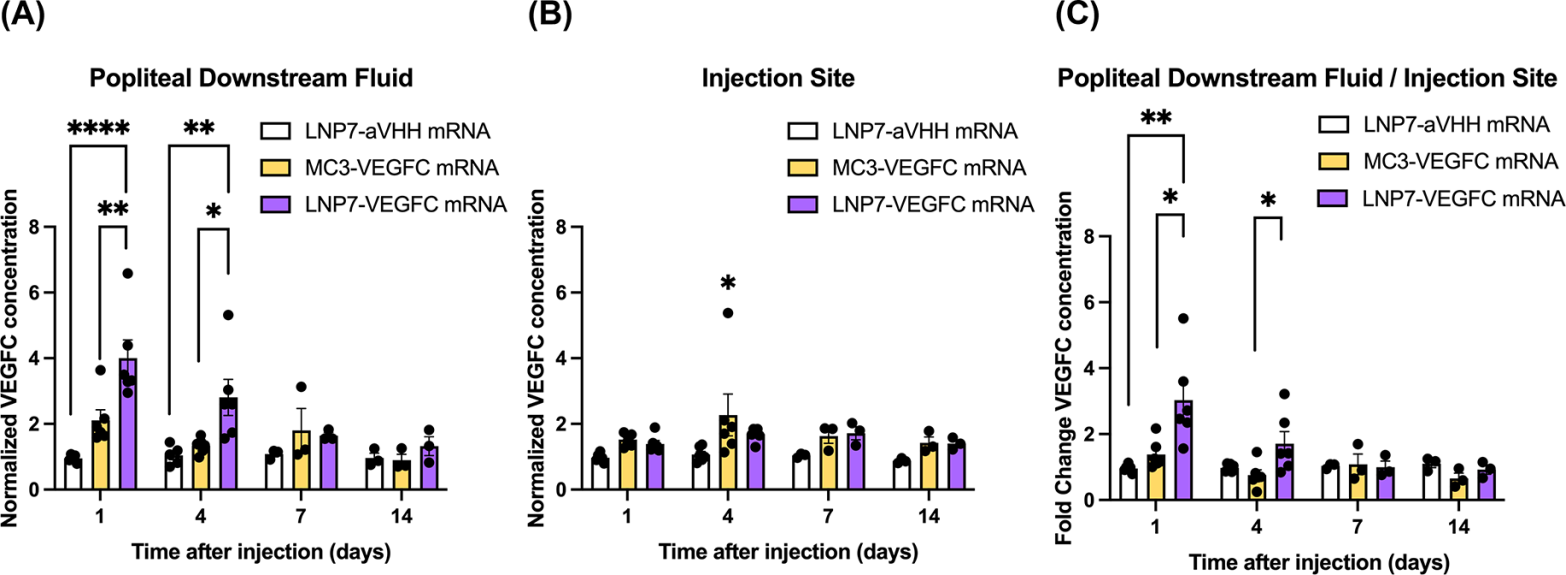
LEC-targeting LNP encapsulating VEGFC mRNA promotes higher VEGFC secretion in the popliteal downstream region. Isolated popliteal dermis and subcutaneous tissues were partially digested, and their popliteal downstream fluid was collected. VEGFC concentration in the downstream fluid was measured with ELISA, and its optical density values were normalized to the LNP7-aVHH mRNA group. Downstream fluid in the LNP7 group contained higher VEGFC without showing a significant difference in VEGFC secretion at the injection site (paw). One-way ANOVA: Tukey test for (A) and (B), and Kruskal–Wallis test was used (C) for multiple comparisons. * for *p* < 0.0332, ** for *p* < 0.0021, **** for *p* < 0.0001, respectively.

Overall, the LNP7-VEGFC mRNA exhibited higher VEGFC secretion in the popliteal downstream fluid (Figure 7A,C). In contrast, the injection site showed no significant differences in VEGFC secretion across all groups (Figure 7). Consistent with the trend observed in the ear (Figure 6), these results indicate that different LNP formulations produce distinct VEGFC concentration profiles at distal and local regions. Notably, VEGFC concentrations at the injection site did not differ between those of the VEGFC mRNA-LNPs (LNP7 and MC3) and even the LNP7-aVHH mRNA control. This suggests that LEC uptake of LNP7 downstream of the injection site—rather than local uptake or interaction at the injection site—was the dominant mechanism responsible for the improved efficacy of VEGFC mRNA delivery by LNP7. While we cannot exclude the possibility that nonlymphatic endothelial cells (e.g., fibroblasts or dendritic cells) also take up VEGFC mRNA-LNPs at the injection site and secrete VEGFC into downstream fluid, the higher VEGFC levels observed with LNP7 compared to MC3 in the popliteal region, coupled with the absence of significant differences at the injection site, support the conclusion that lymphatic endothelial uptake is an important mechanism.

Thus, LNP7 would be expected to generate a stronger VEGFC gradient downstream, particularly at sites of injury. Regarding temporal dynamics, the popliteal downstream fluid in the VEGFC mRNA-LNP7 group peaked at 1 day after injection and was significantly elevated up to 4 days after injection (Figure 7).

### Optimization of LNP VEGFC mRNA Dose for Enhancing Lymphatic Repair and Function

After identifying several LNP formulations with high lymphatic delivery when administered intradermally compared with free mRNA and MC3-based LNPs, we next investigated whether LNP7 would improve the potential therapeutic efficacy of VEGFC mRNA delivery for lymphatic regeneration and restoration of lymphatic pump function. We hypothesized that local VEGF-C mRNA delivery using LNP7 to LECs within vessels draining the site of injury would improve therapeutic outcomes compared to no treatment, free VEGF-C mRNA, or delivery with VEGF-C mRNA with an LNP with poor LEC delivery. To test this, we decided to utilize a mouse tail lymphatic injury model previously developed by our lab, where one chain of lymphangions is damaged while the parallel lymphangion chain on the adjacent side of the tail remains intact.

First, we determined the potential effect of varying doses of LNP7-loaded VEGFC mRNA delivery on lymphatic function and regeneration after lymphatic injury. We used 4 different dosages of VEGFC mRNA (0.04 μg, 0.2 μg, 1 μg, and 5 μg) loaded into LNP7 and LNP7 without VEGFC mRNA (LNP7 loaded with aVHH mRNA instead of VEGF-C mRNA) to serve as a control. Mice were intradermally administered a single injection of LNP7 at the respective dose into the tail 3 days after injury, a few days after injury, but before swelling can be detected in this injury model.

To analyze how lymphatic transport in the intact collecting vessel changed over time after treatment, we utilized NIR imaging to quantify functional metrics of lymphatic contractility both before injury and 7 days after surgery in each treatment group. Administration of 5 μg significantly increased the packet frequency of lymphatic contraction, while lower VEGFC mRNA doses (namely, 0.04 μg, 0.2 μg, and 1 μg) had no significant effect on lymphatic function (Figure 8).

**Figure 8:**
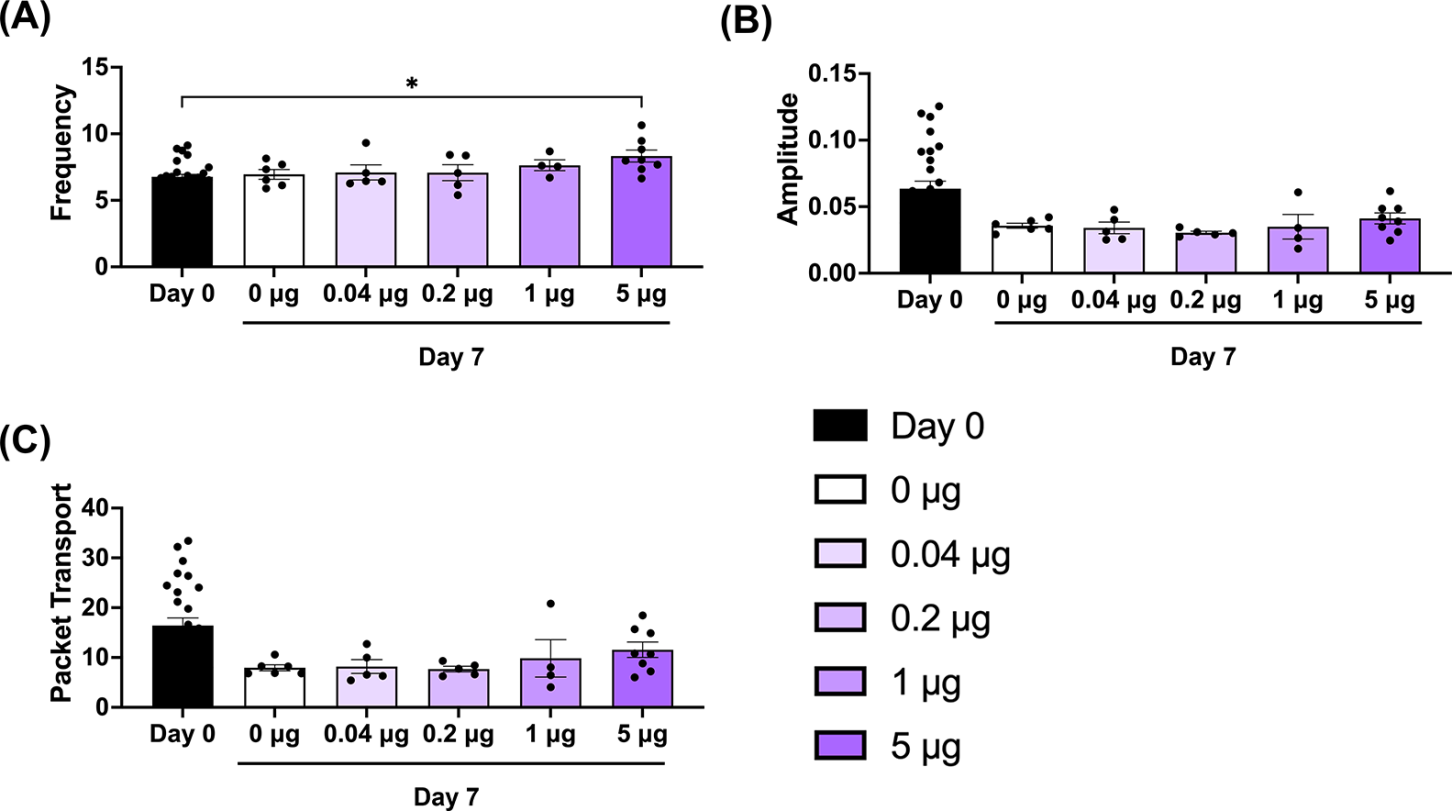
LNP7 loaded with 5 μg VEGFC mRNA significantly increased LV pumping frequency 7 days after mouse tail lymphatic injury *in vivo*. (A) Frequency, (B) amplitude, and (C) packet transport for LNP7-aVHH mRNA (white), 0.04 μg (light purple), 0.2 μg (purple), 1 μg (dark purple), and 5 μg (darker purple) mRNA-LNP7 treatment measured 7 days postsurgery. Each data point corresponds to an independent experiment (*N*_aVHH_ = 6, *N*_0.04 μg_ = 5, *N*_0.2 μg_ = 5, *N*_1 μg_ = 4, and *N*_5 μg_ = 8), and error bars indicate the corresponding SEM. Solid lines above the plots indicate a pairwise comparison for significance using one-way ANOVA with Tukey’s multiple comparisons test with *p* < 0.05 (*), *p* < 0.01 (**), and *p* < 0.001 (***).

We next evaluated lymphangiogenesis by investigating histological changes in the tail. Circular cross-sections were taken from the wound site and the distal portion of the tail from blank and 5 μg treatment groups and stained for podoplanin (PDPN), an LEC marker, and EdU to measure cell proliferation. We demonstrated the presence of PDPN-positive and EdU-positive cells in both the wound and distal sites in the 0 μg of VEGFC mRNA (aVHH mRNA-LNP7) and 5 μg of VEGFC mRNA (VEGFC mRNA-LNP7) groups (Figure 9A). Upon quantification, we observed higher PDPN/EdU colocalization only in the wound site (where lymph leakage occurs) of mice treated with 5 μg of VEGFC mRNA-LNP7 (Figure 9B). Quantification of total PDPN^+^ LECs revealed no significant differences between groups, although VEGFC mRNA-LNP7 treatment showed a slight increase (Figure 9C). This finding indicates that the observed increase in PDPN/EdU colocalization reflects enhanced proliferation rather than a reduction in the number of nonproliferating LECs.

**Figure 9:**
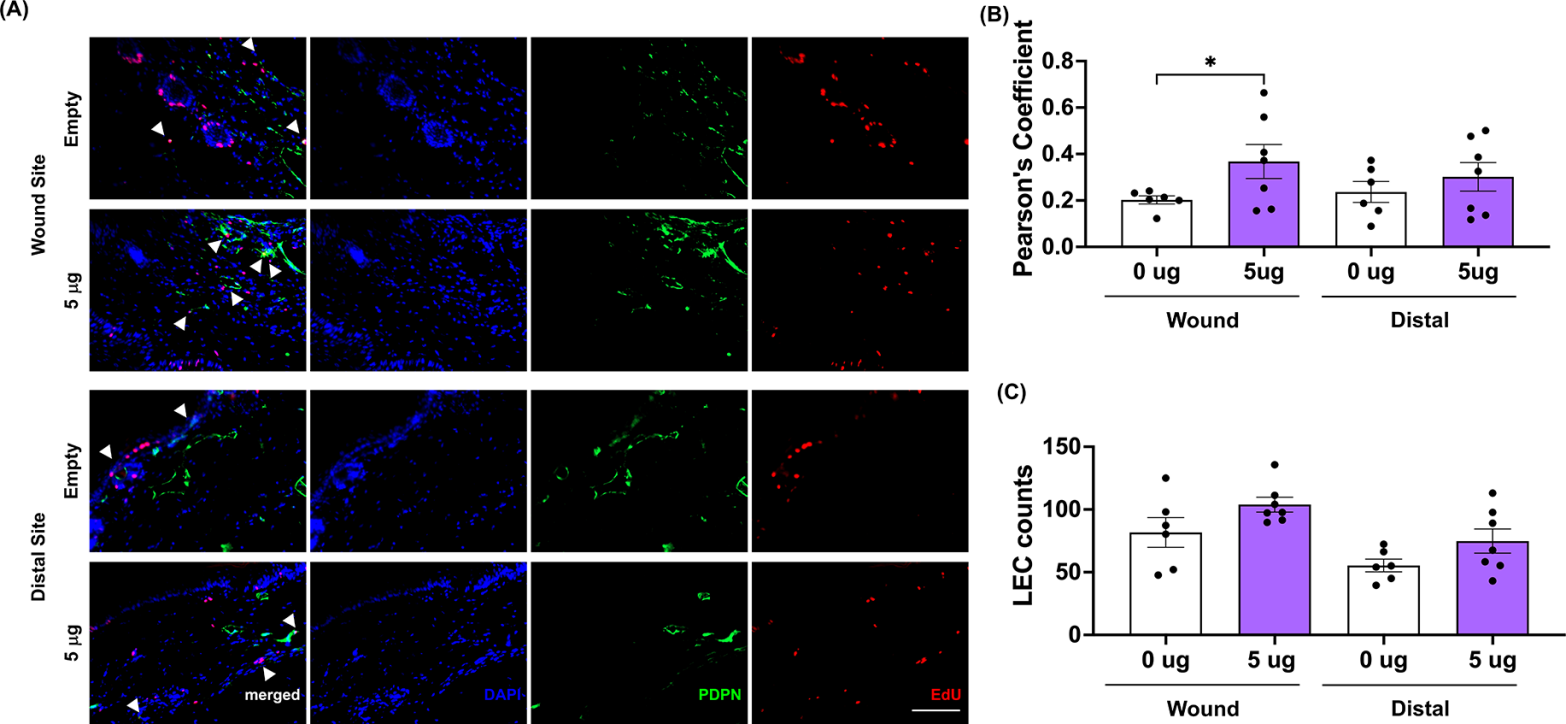
VEGFC mRNA overexpression significantly increases PDPN/EdU colocalization at the wound site 7 days after lymphatic injury. (A) Representative images of merged DAPI (blue), PDPN (green), and EdU (red) in tail sections for the LNP7-aVHH mRNA and 5 μg VEGFC mRNA treatment group at the wound and distal sites 7 days postsurgery. Arrows indicate EdU and PDPN double-positive LECs (20× objective; scale bar = 100 μm). Contrast was enhanced postacquisition for ease of viewing and was performed identically across all images. (B) Pearson’s coefficient and (C) LEC counts in LNP7-aVHH mRNA (white) and 5 μg (purple) groups 7 days postsurgery in the wound and distal sites, measuring the correlation between podoplanin and EdU staining and the count of PDPN^+^ cells within each section. Each data point corresponds to the average of multiple sections (2–3 sections/mouse) taken from each independent experiment (*N*_aVHH_ = 6 mice and *N*_5 μg_ = 7 mice), and error bars indicate the corresponding SEM. Solid lines above plots indicate a pairwise comparison for significance using a nested one-way ANOVA with Tukey’s multiple comparisons test with *p* < 0.05 (*).

### LNP7 Delivery of VEGF-C mRNA Improves Lymphatic Function up to 14 Days after Injury

After demonstrating that a VEGFC mRNA dosage of 5 μg increases lymphatic pump function and lymphangiogenesis 4 days after administration (7 days after injury), we administered this same dose to determine the persistence of improvement on lymphatic pump function and the benefit compared to that of an LNP that does not target LECs. Mice received either LNP7 loaded with aVHH mRNA (LNP7-aVHH mRNA), 5 μg of MC3 loaded with VEGFC mRNA (MC3-VEGFC mRNA), or 5 μg of LNP7 loaded with VEGFC mRNA (LNP7-VEGFC mRNA) into the tail.

Lymphatic transport metrics were measured presurgery and 7 and 14 days postsurgery. Although both MC3-VEGFC mRNA (8.7302 ± 0.2538/s) and LNP7-VEGFC mRNA (9.7136 ± 0.3300/s) therapies improved lymphatic contractile activity compared to LNP7-aVHH mRNA (7.6732 ± 0.2194/s) treatment 7 days postinjury, only VEGFC mRNA-LNP7 showed a significant improvement compared to the aVHH control at 14 days postinjury (Figure 10A). Thus, LNP7-VEGFC mRNA treatment (9.6949 ± 0.2078/s) increases lymphatic function by increasing LV contraction frequency compared to the LNP7-aVHH mRNA (8.8629 ± 0.3432/s) and MC3-VEGFC mRNA (7.9016 ± 0.3530/s) treatments 14 days postinjury (Figure 10A–D) implying that contraction frequency increased by 22.7% with LNP7-VEGFC mRNA compared to the aVHH control, whereas MC3-VEGFC mRNA increased contraction frequency by 12.2% relative to the aVHH control.

**Figure 10:**
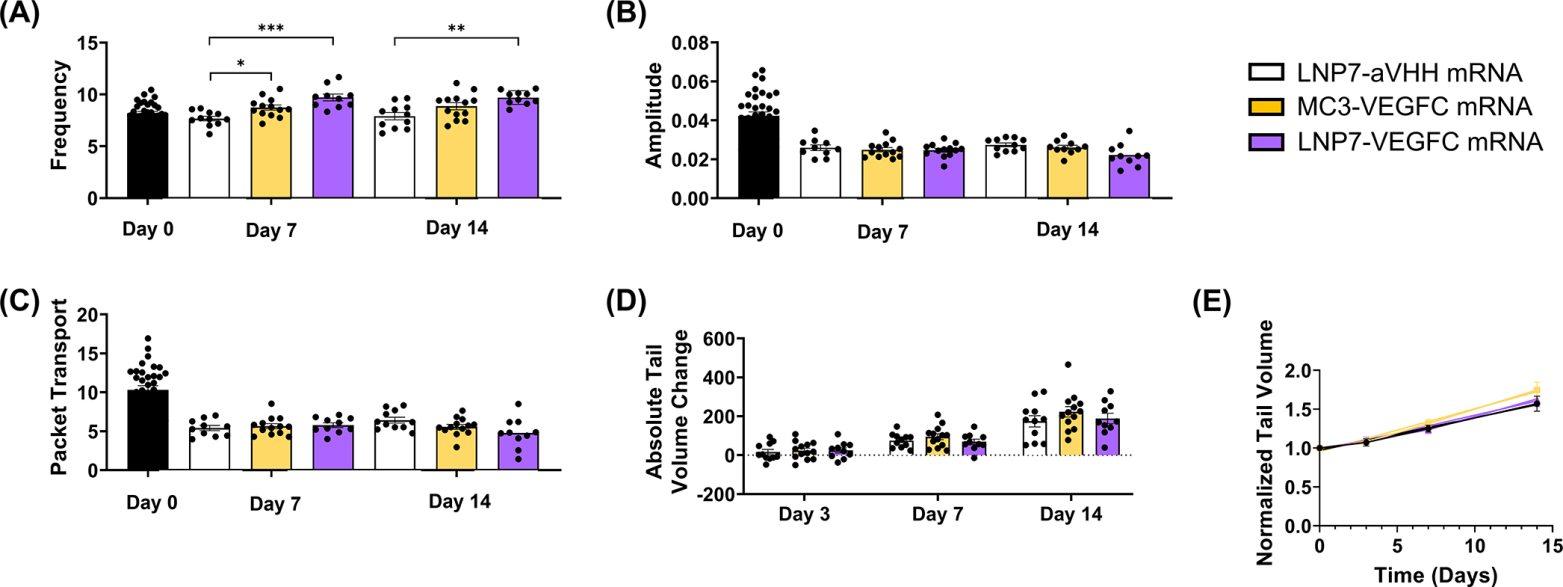
LNP7 loaded with VEGFC mRNA significantly increased LV pumping frequency 14 days after mouse tail single LV ligation surgery *in vivo*. (A) Frequency, (B) amplitude, (C) packet transport, (D) absolute tail volume change, and (E) normalized tail volume for the LNP7-aVHH mRNA (white), VEGFC mRNA-MC3 (5 μg) (gold), and VEGFC mRNA-LNP7 (5 μg) (purple) 14 days postsurgery. A linear regression model was fit to the data to find the best-fit value of the slope and intercept (*y* = β_1_ *x* + β_0_: *y*_aVHH_ = 0.04183 × *x* + 0.9722, *y*_MC3/VEGFC_ = 0.05497 × *x* + 0.9562, *y*_LNP7/VEGFC_ = 0.04563 × *x* + 0.9662). Each data point corresponds to an independent experiment (*N*_aVHH_ = 11, *N*_MC3/VEGFC_ = 13, and *N*_LNP7/VEGFC_ = 10), and error bars indicate the corresponding SEM. Solid lines above the plots indicate a pairwise comparison for significance using mixed-effects analysis with Tukey’s multiple comparisons test and robust regression and outlier removal (ROUT) method to identify and remove outliers with *p* < 0.05 (*), *p* < 0.01 (**), and *p* < 0.001 (***).

Next, we examined the effect of the treatments on the tail swelling. To do so, we used the tail images obtained 3, 7, and 14 days postsurgery. Modeling the tail as a series of truncated cones (36), we calculated total tail volume and determined the corresponding percentage of tail volume change. None of the treatments led to statistically significant changes in tail swelling (Figure 10E). In addition, after fitting a linear regression model (*y* = β_1_ *x* + β_0_), we found no difference in the swelling rate of mice receiving LNP7-aVHH mRNA, MC3-VEGFC mRNA, or LNP7-VEGFC mRNA treatments (β_1, aVHH_ = 0.04183, β_1, MC3/VEGFC_ = 0.05497, and β_1, LNP7/VEGFC_ = 0.04563) (Figure 10E). Therefore, the use of LNP7-VEGFC mRNA is not sufficient to reduce swelling over this time course in this particular animal model. This aligns with our previous reports and is likely due to the presence of an intact outflow route from the tail, minimizing the impact of the surgery on swelling.^70^

Given the benefit of VEGFC treatment on lymphatic function, we next sought to determine if there was any effect on lymphangiogenesis by investigating histological changes in LECs in the tail. Cross-sections were isolated from the wound site and the distal portion of the tail from each treatment group and stained for PDPN and EdU. As in the dose optimization study, we demonstrated the presence of PDPN-positive LECs in both the wound and distal sites in the three treatment groups (Figures 11A and S8). Neither MC3-VEGFC mRNA nor LNP7-VEGFC mRNA treatment significantly modified the lymphatic network density 14 days after mouse tail single LV surgery in the wound or distal sites *in vivo* (Figure 11B–E).

**Figure 11:**
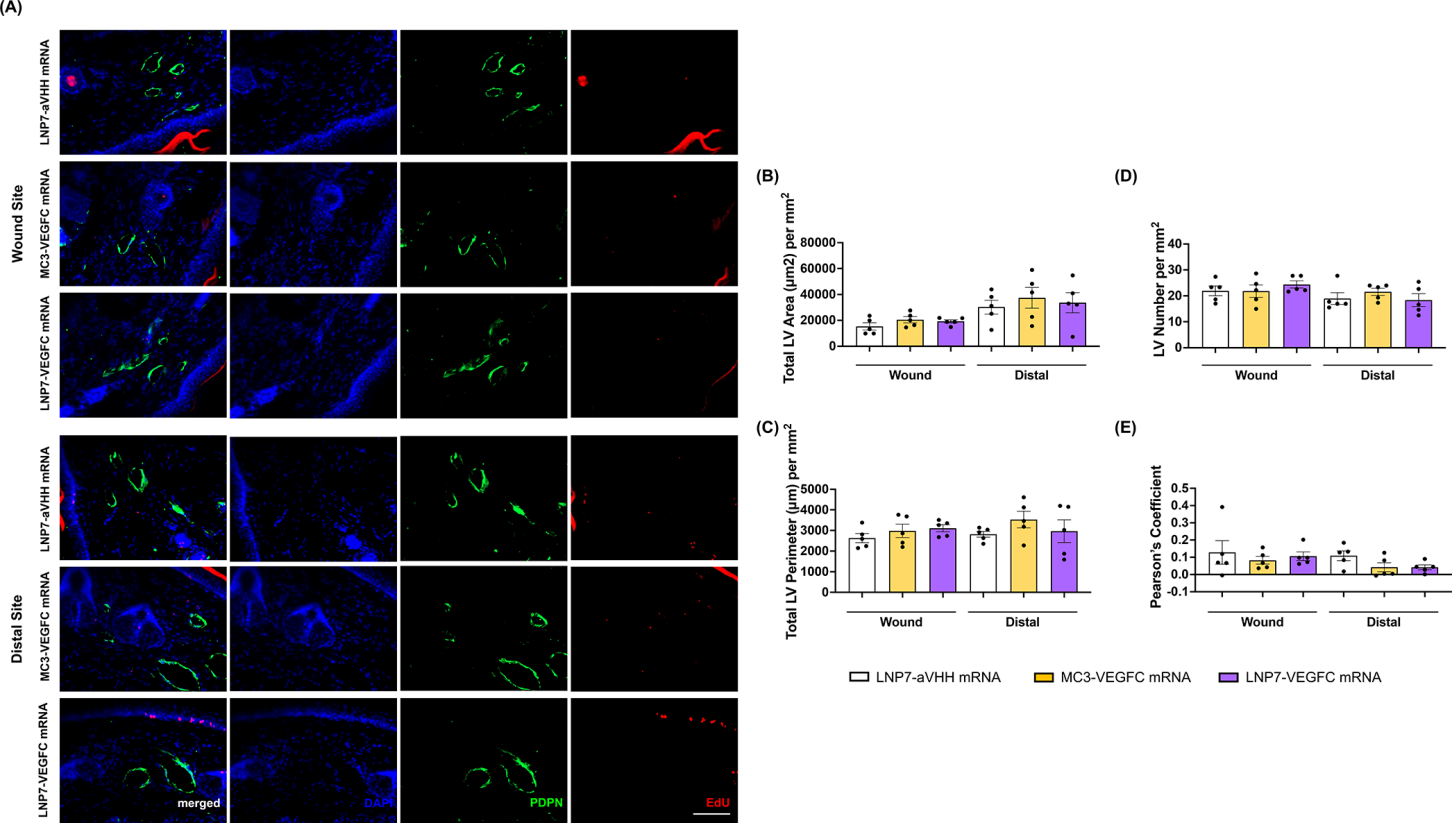
VEGFC mRNA delivery does not affect LV density or PDPN/EdU colocalization 14 days after mouse tail single LV ligation surgery *in vivo*. (A) Representative images of merged DAPI (blue), PDPN (green), and EdU (red) in LVs for the LNP7-aVHH mRNA, VEGFC mRNA-MC3 (5 μg), and VEGFC mRNA-LNP7 (5 μg) in wound and distal sites 14 days postsurgery. (20× objective; scale bar = 100 μm). Contrast was enhanced postacquisition for ease of viewing. Quantification of the (B) total LV area, (C) total LV perimeter, (D) total LV number per mm^2^, and (E) Pearson’s coefficient for the LNP7-aVHH mRNA (white), VEGFC mRNA-MC3 (5 μg) (gold), and VEGFC mRNA-LNP7 (5 μg) (purple) 14 days postsurgery in the wound and distal sites measuring the correlation between podoplanin and EdU staining within each section. Each data point corresponds to the average of each independent experiment (*N*_aVHH_ = 5, *N*_MC3/VEGFC_ = 5, and *N*_LNP7/VEGFC_ = 5), and error bars indicate the corresponding SEM. No significant differences were detected by pairwise comparison for significance using a nested one-way ANOVA with Tukey’s multiple comparisons test with *p* < 0.05.

The lymphatic system is part of the circulatory system,^1^ regulating tissue fluid balance and uptake. Failure to establish adequate tissue drainage results in lymphedema,^69–71^ a condition for which there are currently no curative therapies.^72^ Despite the role of the lymphatic system in many pathologies, enhancing lymphatic drainage *in vivo* through targeted therapy has received little attention.

There have been increased efforts to utilize the lymphatic system as a therapeutic modality for various pathological conditions.^73^ The combinatorial examination of the molecular mechanisms that govern lymphangiogenesis and key factors that dictate lymphatic function, such as lymphatic drainage and pumping, could be applied toward efficient therapies. Here, we proposed the use of an innovative technology for *gene modulation* targeted at LECs to (i) screen and identify LNPs that target LECs, (ii) deliver functional mRNA into lymphatics, and (iii) utilize functional mRNA delivery for targeted therapy administration toward lymphatic regeneration in lymphedema.

Systemic endocrine therapies have caused vascular regression in endocrine organs and other tissues, necessitating the use of more targeted solutions.^74–76^ Nanomedicine for functional mRNA delivery has emerged as a promising avenue to increase cell- and tissue-specific delivery. In lymphedema, although VEGFC administration is the most widely used and thoroughly investigated therapeutic for lymphatic-associated pathologies (e.g., BioBridge,^77^ Lymfactin^48,49^ ), its efficacy in treating the disease has produced mixed results.^19^ Thus, it seems that the development of tools to specifically target the lymphatic system may provide alternative therapeutic approaches toward the development of an effective lymphedema treatment.

These data support the hypothesis that LEC-LNPs that deliver mRNA to LECs may be a useful therapeutic modality. Given the long-established role of VEGFC in lymphangiogenesis,^78,79^ LNPs carrying VEGFC mRNA to lymphatics suggest a promising avenue for therapeutic purposes. VEGFC mRNA delivery in LECs using LNPs will lead to the transient enhancement of VEGFC;^36^ this could avoid problems associated with the long-term upregulation of VEGFC observed with other therapeutics. For example, permanent upregulation of VEGFC has been shown to promote cancer cell metastasis^80^ and is associated with a leaky and dysfunctional lymphatic vasculature. In contrast, LNP7 delivery of VEGFC mRNA led to enhanced proliferation of LECs at the site of injury downstream of LNP delivery and was transient, occurring at 4 days after LNP treatment (7 days after injury) but not persisting at 11 days after treatment following a single dose. While the study here only investigated the effect of a single dose, LNPs have been redosed every 3 weeks in patients for several years,^81^ suggesting that an optimized repeat LNP dosing schedule could be designed for different therapeutic contexts.

VEGFC is an ideal therapeutic cargo to showcase the utility of LEC-targeting LNP therapy because of its role in regulating lymphangiogenesis^78,79^ and its established effects on lymphedema.^82^ Also, the custom-made VEGFC mRNA used for our experiments has previously been demonstrated to induce lymphatic growth and the formation of a functional lymphatic network, restoring lymphatic function without adverse events in a mouse model of lymphedema.^83^

Here, the versatility of SANDS facilitated the identification of LEC-LNPs for functional targeted mRNA delivery. Our ability to identify LNPs that target LECs that reside in LNs and line collecting LVs may revolutionize targeted therapy in multiple pathological conditions. While large library screens were performed with lymph node LECs due to the much larger numbers of LECs that line the subcapsular sinus than that of the collecting vessels in the mouse, validation of the lead candidate LNP7 in collecting LECs provides evidence that properties favorable for uptake by lymph node LECs are similar to those in collecting vessels. Combining LNP screening with scRNA-seq^84^ could be an interesting future endeavor to evaluate whether properties favorable for LEC delivery differ by various LEC subsets.^85^ The simultaneous high uptake of LNP7 by dendritic cells (cDC2 and cDC1), which have been shown to regulate immune responses during lymphedema progression,^86^ provides another cellular target that can be achieved with LNP7 mRNA delivery. In the context of this study, the delivery of VEGFC mRNA to these “off-target” cells did not appear to have any obvious unwanted therapeutic effects. In fact, it is unclear to what extent LEC-delivered vs DC-delivered mRNA delivery of VEGFC mRNA is ultimately responsible for the functional benefit of LNP7. In the context of the injury model, analysis was limited to areas that are prenodal to the LNs that drain the tail; however, it is possible that DCs at the injection site or within the LV itself could also be producing VEGF-C due to LNP mRNA delivery to these cells.

Additionally, the ability of LNP7 to carry mRNA cargoes of different sizes (200 kDa [aVHH mRNA: 231 kDa] to 700 kDa [VEGFC mRNA: 703 kDa]) demonstrates the versatility of this delivery vehicle. Here, we proposed the use of VEGFC mRNA toward a therapeutic effect for lymphedema, but the options for potential cargos are limitless. For example, future work could combine the delivery of mRNA for both VEGFC and its LEC-specific receptor VEGFR3.^87^ Another approach would be to load LNPs with both mRNA and small molecules, such as the immunosuppressive drug Cyclosporine A (CsA), which assists in controlling LEC proliferation and migration^88^ for the restoration of lymphatic function and ultimately the efficient treatment of lymphedema. We also used LNPs loaded with fluorescent cargo for diagnostic purposes. There are endless possibilities, and we hope that our work will be the first of many studies to follow.

In the single-vessel ligation mouse tail model, VEGFC-LNP delivery produced a notable functional improvement with a 22% increase in contraction frequency following LNP7-VEGFC mRNA treatment. However, because one vessel remains intact in this model, swelling is less aggressive, and lymphatic regeneration is not required for resolution. As a result, VEGFC-LNP treatment did not provide a measurable benefit in reducing swelling.^36,89^ This is consistent with other therapeutic studies in this model, where benefits to lymphatic contraction were observed without the therapy speeding up the resolution of swelling. Future studies in models in which edema does not spontaneously resolve will be necessary to determine whether the observed increase in contraction frequency translates into a meaningful therapeutic effect.

To further optimize mRNA delivery for lymphedema treatment, several strategies that have previously been reported could be considered to perhaps enhance the efficacy of LNP7. Potential approaches include active targeting by conjugating a PDPN antibody to LNPs via PEG or increasing the PEG content to 6% to improve circulation and lymphatic uptake^. *^63,67^*^ Additionally, while the effects here were only investigated in the context of a single injection, it could be that repeat injections, or the timing of injections, need to be optimized to realize the full therapeutic potential. Lastly, the swelling in this mouse model of lymphatic injury is known to resolve on its own given enough time, and thus, an animal model involving lymph node dissection and radiation that shows irreversible swelling may be better suited to leverage the regenerative capacity of this therapy.

One limitation of the study is that different lead LEC-LNPs seem to have a preferential uptake by different LNs; for example, LNP3 more efficiently targets LECs in BLNs than LECs in PLNs. Thus, LNP7 is not likely a “one-LNP-fits-all” delivery method. We anticipate that different LNPs would lead to the highest uptake in different areas of interest; thus, designing and characterizing LNPs specifically for the area of interest would lead to the best results. In addition, our reliance on lymph node LECs could result in an LNP candidate with exceptional prenodal LEC targeting being taken up by LECs in the collecting lymphatic before it reaches the lymph node and thus not showing up as a candidate in the screen. Thus, performing a screen in a primate or other large animal model, where much larger amounts of collecting lymphatic vessel tissue could be collected, could provide a better approach that does not rely on the LN.

Another limitation is the observed LNP batch variability in tissue targeting. Varied dialysis and centrifugation methods can alter LNP stability; batch-to-batch consistency is an ongoing area of research in the LNP field. This method may require the need for “fresh” LNPs synthesized the day of injection, as this constraint was built into the experimental design, and the extent that LNP7 would still remain functional after several days of storage and what those ideal storage conditions would be remain outside the scope of this current study.

## Conclusions

In conclusion, the *in vivo* screening of LNPs to identify an LEC-LNP seeks to establish a technique for cargo delivery to the lymphatics. The development of a versatile tool targeting LECs could revolutionize targeted therapy in a variety of disease processes associated with the lymphatic system. Targeted therapy utilizing an LEC-LNP is, *to our knowledge, the first attempt* toward efficient mRNA delivery in LECs and its corresponding use as a therapeutic modality. Specifically, our technique seeks to (i) improve current targeting and delivery efficiency, (ii) reduce costs associated with existing techniques (e.g., antibody conjugation),^90^ and (iii) facilitate the development of lymphatic-specific therapies. We developed a minimally invasive, LEC-targeted, and efficient method to trigger lymphatic regeneration that might present a promising therapeutic modality toward multiple pathological conditions.

## Methods/Experimental

### Animal Studies

All animal experiments were performed following the protocols evaluated and approved by the Georgia Institute of Technology IACUC Review Board (Ethics Approval Number: A100293). Female C57Bl/6 mice aged 7 to 12 weeks (Jackson Laboratory, Bar Harbor, ME) were used for all animal studies. The sexual disparity is due to the disproportional occurrence of lymphatic injury in females because of the dominant occurrence of secondary lymphedema in breast cancer patients and a higher incidence of primary lymphedema in females.^91,92^ Animal weight was recorded before and after all procedures. Studies were carried out at the Physiological Research Laboratories, Georgia Tech, Atlanta, GA.

### mRNA Design and Production

aVHH^57^ and VEGFC mRNAs^83,93^ were designed and produced based on previous studies. The aVHH plasmid was ordered from DNA Geneblock and linearized with Not-I HF (New England Biolabs), then PCR purified using a PCR cleanup kit (Qiagen). Transcribed aVHH mRNA was capped with RNA and added with a poly-A tail following the mScript kit instructions. The purification of aVHH mRNA was performed using the RNeasy kit (Qiagen) and treated with Antarctic Phosphatase (New England Biolabs) for 1 h.

For VEGFC mRNA production, a plasmid encoding codon-optimized mouse Vascular Endothelial Growth Factor C was linearized, and then an *in vitro* transcription reaction was performed using T7 RNA polymerase (Megascript, Ambion). The plasmid encoded a 101-nucleotide-long poly­(A) tail. N1-methylpseudouridine (m1Ψ)-5′-triphosphate (TriLink) instead of Uridine-5′-triphosphate (UTP) was incorporated into the VEGFC mRNA. VEGFC mRNA was capped by using CleanCap (TriLink) and cellulose-purified as described.^93^ All mRNAs were analyzed by agarose gel electrophoresis, measured for concentration, and stored frozen at −20 °C.

### Nanoparticle and mRNA Dosing

Before injection, all LNPs loaded with aVHH, Cy3-labeled microRNA hairpin (Horizon; IP-004500-01-50), or VEGFC mRNA were characterized as described in detail below (Figure S9). Animals were anesthetized using inhaled isoflurane (5% induction, 2–2.5% maintenance). In this study, all LNPs were injected intradermally based on initial screenings, which showed higher uptake by LECs compared to intravenous administration (Figure S10). During LNP screening and follow-up validation studies with normal mice, we injected LNPs in each paw of the mice with an mRNA dose of 1.5 mg/kg intradermally. In each screening run, the number of mice was 3 and 2 for LNPs and saline, respectively.

To determine the dose response of LNP7, lymphedema-induced mice by single LV surgery were injected with a single injection intradermally into the tip of the tail with varying dosages of VEGFC mRNA (0.04 μg, 0.2 μg, 1 μg, and 5 μg)^83^ on day 3 postsingle LV ligation surgery. Control animals (labeled as 0 μg in Figure 8) were intradermally injected into the tip of the tail with aVHH-loaded LNPs at a dose of 5 μg of aVHH to match the LNP dose of the maximum VEGF-C mRNA dose that was delivered. The number of mice was 6, 5, 5, 4, and 8 for 0 μg, 0.04 μg, 0.2 μg, 1 μg, and 5 μg VEGFC mRNA-LNP7, respectively.

To monitor the therapeutic effect on lymphatic function by VEGFC mRNA-LNP7, lymphatic injury-induced mice by single LV surgery were injected intradermally at the tip of the tail with empty LNP7 and 5 μg (mRNA dosage) of VEGFC mRNA-LNP7 on day 3 postsingle LV ligation surgery. The numbers of mice were 6 and 7 for saline and 5 μg of VEGFC mRNA-LNP7, respectively. The difference in number between groups for the various lymphatic injury experiments was due to some mice having to be withdrawn from the study due to IACUC endpoint criterion from poor tissue healing in response to the surgery, which occasionally occurs due to the artery accidentally being injured during cauterization of the tail wound. Mice were randomized after injury to determine which therapeutic treatment they would receive.

### Tissue Collection

For LNP screening and characterization, tails, LNs, and LVs were isolated 12–16 h after nanomedicine administration (Figure S11). During lymphedema studies, tails were collected 7 and 14 days postinjury for all treatment groups. For each tail, two 1 cm-long tissue samples were harvested at the wound and distal to the site of injury. Harvested tails were fixed in 10% neutral buffered formalin (3800600; Leica Biosystems, Wetzlar, Germany) and cryo-sectioned into 10 μm sections.

### Tissue Dissociation for Flow Cytometry and SANDS

Isolated LNs and LVs were washed in 1 mL of PBS (21-030-CV; VWR International) on ice. LNs were added to 500 mL of Collagenase D solution (1 mg/mL in PBS; 11088866001; Sigma-Aldrich)/DNase I (40 μg/mL in PBS; 10104159001; Sigma-Aldrich) and incubated for 1 h at 37 °C on a rocker/vortex at 300 rpm. LVs were added in 500 μL of Dispase II/Collagenase I mixture (Dispase II (50 mg; 4942078001; Sigma-Aldrich), Collagenase I (20 mg; 17-100-017; Thermo Fisher Scientific), and BSA (0.1 g; A7906; Sigma-Aldrich) in 10 mL of DMEM (11039-047; Thermo Fisher Scientific). The cell suspension was passed through a 70 μm strainer (07-201-431; Thermo Fisher Scientific). Any remaining tissue samples were gently disrupted using a syringe plunger. Filtered cells were centrifuged at 300 g for 5 min at 4 °C. Suspended cells were then used for subsequent experiments.

### Flow Cytometry

Suspended cells prepared as above were stained for live/dead cell quantification with the Zombie NIR Fixable Viability Kit following the manufacturer’s protocol (1:100 dilution, 423111; BioLegend, San Diego, CA). Subsequently, cells were washed with FACS buffer (10 mg/mL BSA (A7906; Sigma-Aldrich) in PBS). Antibodies were prepared in the FACS buffer, and cells were stained on ice for 30 min in the dark. Information on the corresponding laser, concentration, and vendor information on antibodies used for the flow panel in this study was as follows: (i) Live/Dead (BV510, 1:100, 423111; BioLegend), (ii) CD31 (AF647, 1:100, 102416; BioLegend), (iii) PDPN (AF488, 1:100, 156208; BioLegend), (iv) aVHH (APC, 1:100, A01994; GenScript, Piscataway, NJ), (v) CD45 (PE, 1:100, 147712; BioLegend), (vi) CD54 (PE-Cy7, 1:300, 116122; BioLegend), (vii) CD309 (PER-CP-Cy5-5, 1:100, 121918; BioLegend), (viii) CD11b (BV421, 1:100, 101236; BioLegend), (ix) MHCII (BV650, 1:1500, NBP2-00462; Novus Biologicals, Littleton, CO), (x) CD11c (BV786, 1:10, 117335; BioLegend), (xi) CD64 (BV711, 1:100, 139311; BioLegend), and (xii) F4-80 (APC-Cy7, 1:100, 157315; BioLegend). Compensation controls for antibodies were made using UltraComp eBeads Compensation Beads (01-2222-42; Thermo Fisher Scientific). Data was acquired on the BD FACS Aria III Cell Sorter (BD Biosciences) and analyzed with FlowJo Software. The gating strategy is described in Figures S12 and S13. Relevant cell counts are presented in Tables S1, S2, and S3.

### *In Vivo* EdU Labeling

During lymphatic injury studies, EdU labeling and its detection were followed with the protocol of the Click-iT EdU Cell Proliferation Kit for Imaging Alexa Fluor 594 dye (C10639; Thermo Fisher Scientific). Briefly, 10 mM of EdU was prepared by diluting EdU with 2 mL of DMSO. Twenty-five mg/kg of EdU (25 mg/kg) was injected intradermally into the tail 16 h prior to tail harvest.

### EdU Detection and Immunofluorescence Staining

For antigen retrieval, tail sections were incubated in sodium citrate at 90 °C for 30 min. Sections were permeabilized with 0.5% Triton X-100 (X100–5 ML; Sigma-Aldrich) in PBS at room temperature for 20 min. EdU was detected with Click-iT Reaction cocktail for 30 min at room temperature protected from the light. Afterward, DNA was stained with Hoechst solution for 30 min at room temperature in the dark.

After washing the Hoechst solution on tissues with PBS, nonspecific binding was blocked with 10% goat serum (G9023; Sigma-Aldrich) in PBS for 1 h at room temperature. Slides were probed with hamster IgG monoclonal anti-PDPN (1 mg/mL in PBS; ab11936; Abcam, Cambridge, UK) at 1:100 dilution in PBS solution overnight at 4 °C. The following day, the slides were washed with PBS and incubated in the dark with Alexa Fluor 488-conjugated goat antihamster IgG (2 mg/mL; A21110; Thermo Fisher) at 1:200 dilution in PBS solution for 4 h at room temperature. For nuclei staining and mounting sections, an Invitrogen ProLong Gold AntiFade with DAPI (Thermo Fisher) was used.

A Zeiss Axio Observer fluorescent microscope was used to image slides after staining, and analysis was performed on high-powered sections (20× objective) with at least 5 high-powered fields (hpf) per location (i.e., wound or distal) per mouse.

### BCA Protein Concentration and ELISA for VEGFC Secretion Measurement

The subcutaneous and dermal layers of the popliteal region were harvested, and the paw skin was sonicated for 2 min with RIPA buffer with protease inhibitor (G-Bioscience (VWR); 786-108). Fluids were collected after 5 min of centrifuging at 12,000 × *g* at 4 °C, and total protein concentration was measured using the Pierce BCA Protein Assay Kit (Thermo Fisher 23250). VEGFC secretion at specified time points (1-, 4-, 7-, and 14-days postinjection) of tissues was measured using the VEGFC Rat ELISA Kit (Invitrogen, BMS626-2) following the manufacturer’s instructions. The total amount of protein loaded into each well was 30 μg. Optical density values were normalized to those of the LNP7-aVHH mRNA group.

### Whole-Mount Staining and Confocal Imaging

After harvest, the posterior ear skin was exposed to the face and fixed with a 4% paraformaldehyde solution (PFA; Electron Microscopy Science 15710). To determine lymphatic growth and LEC proliferation, immunofluorescent staining was performed using anti-PROX1 (1:200; ab199359), anti-PDPN (1:100; ab11936), and NucBlue (Invitrogen; R37606) to label LECs. After the whole-mount assay, the posterior ears were imaged at 5× and 20× using a confocal laser scanning microscope (Zeiss LSM 980 NLO). Raw z-stacked confocal images were maximum-intensity projected and postprocessed using ImageJ and Fiji, software developed by the National Institutes of Health (NIH), to reduce background noise and enhance image contrast.

### Data Preparation, Visualization, and the Application of Machine Learning Models on the SANDS Library

SANDS sequencing data, which included lipid types and their corresponding molar ratios of each LNP, was used for the analysis. Since Screening 2 data due had a very low number of aVHH+ LECs (Figure S2), it was not moved on for sequencing of barcode reads. In addition, non-QC-passed LNPs (>200 nm) were not included in the dataset. One-hot encoded categorical lipid type variables and molar ratios were stored as input features. The product of normalized PLN, ALN, and BLN barcode readouts, generated using a custom-built Python script for SANDS analysis, was used as the label representing total LN LEC delivery.

For visualization, a histogram of total lymph node LEC delivery counts was generated to characterize the distribution of delivery efficiencies across all of the LNP candidates. The *x*-axis represents the total LN LEC delivery. A log scale was applied to the *x*-axis to accommodate the wide dynamic range of delivery values. A threshold of 100 total LN LEC deliveries was selected to differentiate candidates with high LEC targeting from those with lower performance. The threshold was visualized as a vertical dashed red line. Histogram bins were colored to highlight candidates exceeding this threshold and annotated with the identifiers of the selected LNPs.

Uniform Manifold Approximation and Projection (UMAP) was performed to visualize similarities in lipid nanoparticle composition across the tested formulations.^94^ Prior to dimensionality reduction, input features were standardized by the z-score normalization. UMAP embeddings were computed using the umap-learn Python package (version 0.5.7) with the following parameters: n_neighbors = 5, min_dist = 1, metric = “euclidean”, and random_state = 42.

The dataset was divided into training (80%) and testing (20%) subsets. The training data were used to fit the models, while the testing data were reserved for hyperparameter tuning. Three regression models—linear regression, random forest (RF)^95^ (scikit-learn 1.6.1), and XGBoost (XGB)^96^ (xgboost 2.1.3)—were implemented to predict LNP delivery and determine feature importance. For the RF, the hyperparameters were set to max_depth = 4, n_estimators = 4, and random_state = 42. The XGB model employed: colsample_bytree = 1, learning_rate = 0.01, max_depth = 10, and n_estimators = 2000.

Model performance was evaluated via mean squared error (MSE) and determination coefficient (*R* ^2^) defined at eqs 1 and 2.

1 MSE=∑i=1n(yi^−yi)2n

2 R2=1−∑i=1n(yi−yi^)2∑i=1n(yi−y̅)2

where *n* is the number of data, *y_i_* is the ground truth, y̅ is the mean of the data, and yi^ is the *i* ^th^ prediction.

To interpret model predictions, we compared coefficients from the linear regression model and Shapley values from the tree-based models. In linear regression, the variable importance was determined by ranking the magnitude of the fitted coefficients. For the tree-based models, Shapley additive explanations (SHAP) were used to quantify the contribution of each feature to the model output.^97^ The Shapley value for a given variable represents the average marginal contribution of including that variable across all possible subsets of features (eq 3); this captures not only the magnitude but also the direction of each feature’s influence.

3 $$i\mathrm{’s Shapley value}=\phi_{i}\left(\vartheta \right)=\underset{S\subseteq D\left\{i\right\}}{\sum }\frac{\left|S\right|!\left(\left|D\right|-\left|S\right|-1\right)!}{\left|D\right|!}\left(\vartheta \left(S\cup \left\{i\right\}\right)-\vartheta \left(S\right)\right)$$

### NIR Imaging for Monitoring Lymphatic Function *In Vivo*

NIR lymphatic imaging was performed according to previously published methods.^78^ Before lymphatic vessel imaging, LI-COR IRDye 800CW (929-70021; LI-COR Biosciences, Lincoln, NE) was diluted in DMSO to a concentration of 10 mg/mL. Then, 10 μL of the dye solution was injected intradermally into the tip of the tail.

The lymphatic vessel imaging was recorded with a customized imaging system consisting of a Lambda LS Xenon arc lamp (LB-LS; Sutter Instrument, Novato, CA), an Olympus MVX-ZB10 microscope (Olympus Corporation, Japan), a 769 nm band-pass excitation filter (49 nm full-width half-maximum; FWHM), an 832 nm band-pass emission filter (45 nm FWHM), and an 801.5 nm long-pass dichroic mirror. Images were acquired with a Photometrics Evolve Delta 512 EM-CCD instrument (Teledyne Photometrics, Tucson, AZ). The field of view was centered on the mouse’s tail 7 cm downstream toward the base of the tail from the injection site at the tip of the tail. Animals were imaged continuously from the time of injection until 20 min postinjection with a 50 ms exposure time and a frame rate of 10 fps. Baseline NIR metrics and tail images were collected in all groups prior to surgery (day 0). For the dosage optimization study, NIR functional metrics were again measured after surgery on day 7 prior to euthanasia and tissue collection. When we measured the therapeutic effect of VEGFC mRNA-LNP, NIR functional metrics were measured after surgery on days 7 and 14. Tail volume measurements were taken after surgery on days 3, 7, and 14. Animals were euthanized, and tissue was collected on day 14.

### NIR Analysis for Quantifying Lymphatic Function

Analysis of NIR functional metrics was performed during the steady-state period ranging from 5 to 20 min after injection, as defined previously.^98^ Packets of fluorescence were detected by identifying peaks and troughs in the fluorescence signal over time. These measurements were used to calculate previously reported metrics for this model, such as packet frequency, amplitude, integral, and transport.^98,99^ All data were normalized to the baseline NIR intensity. Sample sizes for each experiment are included in the corresponding figure caption.

### Single LV Ligation Lymphatic Injury Model

To induce lymphatic injury on the mice tail, single LV ligation surgery was performed.^70,99^ Briefly, animals were anesthetized with 5% isoflurane and maintained on 2–2.5% isoflurane during the entire surgery. Prior to surgery, NIR dye was injected intradermally to the tail for the visualization of both the dominant and nondominant LVs. Animals received incisions 1.6 cm from the base of the tail, spanning 80–90% of the circumference of the tail. The nondominant vessel was left untouched, and function was verified after injury via imaging. Animals in which LVs were improperly ligated or blood vessels were damaged were excluded from the study. For tissue collection, animals were euthanized using CO_2_.

### Quantification of Microscopic Images

Images of mouse tails were segmented in ImageJ, and the corresponding diameters and lengths were measured. Total tail volume was calculated by a truncated cone volume equation for each segment, summed. Absolute tail volume change was calculated by subtracting the corresponding tail measurement obtained in all groups prior to surgery (day 0), and normalized tail volume by dividing by this measurement. The sample size for each experiment is included in the corresponding figure caption.

Subsequently, the total LV area, LV perimeter, and LV number per square mm were measured in ImageJ for at least 5 hpf per location (i.e., wound, distal) per mouse. LVs were identified by positive staining for PDPN. LVs were manually selected, and the area and perimeter of each selection were measured. The number of LVs was determined as the number of distinct selections per hpf. For EdU staining, lymphatic-specific proliferation was identified by quantifying the colocalization of PDPN and EdU. Specifically, the ImageJ plugin “*JACoP*”^100^ and the Pearson’s coefficient^101^ were used. A minimum of five specimens was analyzed per condition/tissue sample. Actual numbers of samples are included in the corresponding figure caption.

### Statistical Analysis

To compare lymphatic uptake among the lead LEC-specific LNPs, one-way ANOVA was used, combined with robust regression and outlier removal (ROUT). To compare uptake by LNs and LVs among saline, free aVHH, MC3, and LNP7, two-way ANOVA was used with Tukey’s method to correct for multiple comparisons. To compare uptake among different cell types, an ordinary one-way ANOVA was used. To compare the effect of different dosages of VEGFC mRNA in NIR metrics, one-way ANOVA was used with Tukey’s multiple comparisons correction. To compare the effect of different LNPs loaded with VEGFC mRNA in NIR metrics, mixed-effects analysis was used with Tukey’s multiple comparisons correction. To compare absolute tail volume change, two-way ANOVA with Geisser–Greenhouse correction was used with Tukey’s multiple comparisons correction. To compare the normalized tail volume, a simple linear regression model was used. All histological measurements were compared between groups by nested one-way ANOVA with Tukey’s multiple comparisons test after ROUT to remove outliers within an individual specimen and tissue location. An unpaired *t*-test was used to compare different types of administration. Each data point corresponds to either an independent experiment or the average of each corresponding condition, as stated in the figure caption. Data were analyzed by using GraphPad Prism 7 (GraphPad Software, San Diego, CA). Reported *p*-values are multiplicity adjusted to account for multiple comparisons. For all cases, significance was defined as *p* < 0.05 (*), *p* < 0.01 (**), *p* < 0.001 (***), or *p* < 0.0001 (****).

### LNP Formulation

Nucleic acids (mRNA and DNA barcodes) were diluted in 10 mM citrate buffer, pH 3, while lipomer, PEG, cholesterol, and helper lipids were diluted in ethanol. The compounds and their molar ratios for the lipid phase of the lead LEC-specific LNPs and MC3-based LNPs (MC3) were listed in Table S1.^69^ LNPs were formulated by injecting the citrate and lipid phase into a microfluidic device as previously described (Figure S14).^56–58^ The flow rates of the citrate and lipid phases are 600 and 200 μL/min, respectively. The syringes (Hamilton Company) for injection to the microfluidic device were controlled by syringe pumps (Harvard Apparatus) programmed using FLOWCONTROL software (Harvard Apparatus). The weight ratio of lipomer to mRNA in this study was maintained at 10:1.

The ionizable lipids used in this study were stereopure lipomers (C12S, C12R, C13S, and C13R were provided from the Dahlman lab), MC3 (555308; MedKoo Biosciences, Inc., Morrisville, NC), and cKK-E12 (BP-29590; BroadPharm, San Diego, CA). All PEG, cholesterol, and helper lipids were purchased from Avanti Polar Lipids (Alabaster, AL).

### LNP Characterization

After LNPs were diluted in sterile 1× PBS to a concentration of ∼0.06 μg/mL, the hydrodynamic diameter (nm), polydispersity (PD), and polydispersity index (PDI) of the LNP were measured using dynamic light scattering (DLS) using DynaPro Plate Reader II (Wyatt Technology). LNPs were included in the experimental design if they met all of the following criteria: (i) diameter >20 nm, (ii) diameter <150 nm, (iii) correlation function with one inflection point, and (iv) PDI < 0.4.

Quality-controlled LNPs were dialyzed with 1× PBS using dialysis cassettes with membranes (87735 and 87734; Thermo Fisher Scientific, Waltham, MA) for 90 min. Dialyzed LNPs were sterile-filtered with a 0.22-μm filter (371-2115-OEM; Foxx Life Sciences, Londonderry, NH).

The nucleic acid concentration of the filtered LNPs was measured using a NanoDrop (ND-ONE-W; Thermo Fisher Scientific). To quantify the nucleic acid concentration inside the LNP, the LNP was loaded in a 96-well plate (675097; Greiner Bio-One), and the RiboGreen assay was performed following manufacturer’s protocol (R11490; Invitrogen, Waltham, MA).

### Zeta Potential

Formulated lipid nanoparticles (LNPs) were diluted in ultrapure water to a final concentration of 100 ng/μL. A total volume of 1 mL of the diluted LNP solution was loaded into a folded capillary zeta cell (DTS1070; Malvern Panalytical). Zeta potential measurements were performed at 25 °C using a Zetasizer Nano ZS (Malvern Panalytical Ltd., Worcestershire, UK). The refractive index (RI) and absorption of the particle material were set to 1.40 and 0.01, respectively. The dispersant (water) was assigned a viscosity of 0.8872 cP and a refractive index of 1.330. Each experiment was measured in triplicate and analyzed under Zetasizer Software version 8.02 (Malvern Panalytical Ltd., Worcestershire, UK).

### SANDS (Species Agnostic Nanoparticle Delivery Screening)

In the screening of LNPs to identify LEC-specific LNPs, SANDS was conducted as previously described.^56^ Briefly, 150 LNPs with varying lipid compositions were formulated with 56-nucleotide-long ssDNA sequences serving as DNA barcodes and aVHH mRNA. Each ssDNA sequence contained a unique 8-bp barcode sequence in its center, and these sequences were purchased from Integrated DNA Technologies. Quality-controlled LNPs were screened in female C57Bl/6 mice via intradermal injection in each paw of the mice at a dosage of 1.5 mg/kg, and aVHH+/podoplanin+ LECs from lymph nodes (ALN, BLN, and PLN) were isolated and sequenced using Illumina Miniseq with primers from Nextera XT adapter sequences. Lymphatic uptake was quantified based on the normalized barcode counts for each LN using a custom Python-based tool.

### Cell Culturing and LNP Transfection

Human dermal LECs were isolated from human foreskin tissue following the protocol published by Rogic and coworkers.^102^ The cells were seeded in a 24-well plate (353047; Thermo Fisher Scientific) at a density of 15,000 cells/well. After 24 h, LNP7 was added with a total aVHH mRNA dose of 4, 20, or 100 ng in eight separate wells.^57^ Six h post-transfection, the media was removed and replaced with fresh media. Cells cultured with physiological endothelial basal medium (EBM; CC-3121; Lonza, Switzerland) with recommended supplements and 10% DMSO (D2650; Sigma-Aldrich) served as negative and positive controls, respectively.

### *In Vitro* Toxicity Study of LNP7 Using Live/Dead Staining and AlamarBlue Assay

The viability of monolayers after treatment was determined using a viability kit (L3224; Thermo Fisher) to distinguish live (Calcein-AM) and dead cells (Ethidium homodimer-1). Staining was performed following the manufacturer’s instructions; cells were incubated with calcein-AM and ethidium homodimer-1 for 20 min at 37 °C. Then, monolayers were rinsed with PBS before imaging on an inverted microscope (AxioObserver.Z1; Zeiss). Tile images of individual wells were acquired using a 1× tube lens and a 2.5× objective (Plan-Neofluar 2.5×/0.075 Pol). Zen Black software was used to stitch tile images with a 10% overlap to reconstruct the image of the entire well. Images were then processed by using ImageJ (NIH), where the “watershed” function was utilized to segment individual cells. Following processing, the “analyze particle” function in ImageJ was used to count individual cells in the live (green) and dead (red) channels with thresholds set to 0–25 and 0–80, respectively. Viability was further confirmed using the alamarBlue cell viability reagent (DAL1025; Thermo Fisher). According to the manufacturer’s instructions, 1× alamarBlue reagent was added to the cell media of monolayers after treatment and incubated for 6 h at 37 °C. Fluorescence intensity was measured with an excitation of 530 nm and an emission of 590 nm using a plate reader (Synergy H4; BioTek). The average background fluorescence was subtracted, and the individual fluorescence intensity per well was reported.

## Supplementary Material



## Notes

Supplementary figures: outline of total LNPs with their compositions for screens, LEC and LEC/aVHH^+^ counts for screens, SANDS library visualization via histogram and UMAP, relationship between LNP parameters and lymphatic node delivery, coefficient and feature importance analyses for LNP parameter contribution to LEC delivery, lymphatic-specific uptake of LNP7, LNP7 *in vitro* toxicity test, Immunofluorescence controls in tail sections, characterization of MC3 and LNP7 loaded with different mRNA cargos, effect of the route of administration of LNP delivery, schematic of engineer LEC-targeting LNPs, gating strategies for FACS in LNs and LVs, schematics of LNP formulation and quality assessment, compounds and molar ratios of lead LEC-LNP candidates, and average cell counts. Supplementary tables: compounds and molar ratios of lead LEC-specific LNP candidates, and flow cytometry cell counts (PDF)
