# Supplementary material for: Lipid Nanoparticle Library Screen Reveals Lymphatic Endothelial Cell-Targeting Lipid Nanoparticle for Delivering Vascular Endothelial Growth Factor C mRNA after Lymphatic Injury
Source: ACS Nano. 2025 Nov 21;19(48):40873–91. doi: 10.1021/acsnano.5c12080 (PMC12874650; doi:10.1021/acsnano.5c12080)
Supplement: Supplementary file 1 [file nn5c12080_si_001.pdf]

Supporting Information

Lipid nanoparticle library screen reveals lymphatic endothelial cell-targeting lipid nanoparticle for delivering vascular endothelial growth factor C mRNA after lymphatic injury

Eleftheria Michalaki[1], Kiyoung Jeong[2], Rachel Chin[3], Zhiming Qi[2], Lauren Liebman[2], Yarelis González-Vargas[2], Elisa Schrader Echeverri[2], Kalina Paunovska[2], Hiromi Muramatsu[5], Norbert Pardi[5], Beth Jiron Tamburini[6], Zoltan Jakus[7], James E. Dahlman[2], and J. Brandon Dixon[1, 2, 4*]

[1]George W. Woodruff School of Mechanical Engineering, Georgia Institute of Technology; Atlanta, GA 30332, USA.

[2]Wallace H. Coulter Department of Biomedical Engineering, Georgia Institute of Technology and Emory University; Atlanta, GA 30332, USA.

[3]Department of Biology, Georgia Institute of Technology; Atlanta, GA 30332, USA.

[4]Parker H. Petit Institute for Bioengineering and Bioscience, Georgia Institute of Technology; Atlanta, GA 30332, USA.

[5]Department of Microbiology, Perelman School of Medicine, University of Pennsylvania; Philadelphia, PA 19104, USA.

[6]University of Colorado School of Medicine, Department of Medicine, Aurora, CO 80045, USA

[7]Semmelweis University School of Medicine, Department of Physiology, Budapest, 1085, Hungary

*Corresponding author. Email: dixon@gatech.edu.

Supplementary Figures

Screening 1

Name	Lipomer	Cholesterol	PEG	Helper Lipid	Lipomer	Cholesterol	PEG	Helper Lipid
LNP 1	cKK-E12	Cholesterol	C14PEG2K	DSPC	30	30	2.5	37.5
LNP 2	cKK-E12	Cholesterol	C14PEG2K	DSPC	35	18	2.5	44.5
LNP 3	cKK-E12	Cholesterol	C14PEG2K	DSPC	45	42	2.5	10.5
LNP 4	cKK-E12	Cholesterol	C14PEG2K	DSPC	50	35	2.5	12.5
LNP 5	cKK-E12	Cholesterol	C14PEG2K	DSPC	52.5	15	2.5	30
LNP 6	cKK-E12	Cholesterol	C14PEG2K	DSPC	57.5	20	2.5	20
LNP 7	cKK-E12	Cholesterol	C14PEG2K	DOPE	30	30	2.5	37.5
LNP 8	cKK-E12	Cholesterol	C14PEG2K	DOPE	35	18	2.5	44.5
LNP 9	cKK-E12	Cholesterol	C14PEG2K	DOPE	45	42	2.5	10.5
LNP 10	cKK-E12	Cholesterol	C14PEG2K	DOPE	50	35	2.5	12.5
LNP 11	cKK-E12	Cholesterol	C14PEG2K	DOPE	52.5	15	2.5	30
LNP 12	cKK-E12	Cholesterol	C14PEG2K	DOPE	57.5	20	2.5	20
LNP 13	cKK-E12	Cholesterol	C14PEG2K	18:1 CAP PE	30	30	2.5	37.5
LNP 14	cKK-E12	Cholesterol	C14PEG2K	18:1 CAP PE	35	18	2.5	44.5
LNP 15	cKK-E12	Cholesterol	C14PEG2K	18:1 CAP PE	45	42	2.5	10.5
LNP 16	cKK-E12	Cholesterol	C14PEG2K	18:1 CAP PE	50	35	2.5	12.5
LNP 17	cKK-E12	Cholesterol	C14PEG2K	18:1 CAP PE	52.5	15	2.5	30
LNP 18	cKK-E12	Cholesterol	C14PEG2K	18:1 CAP PE	57.5	20	2.5	20
LNP 19	cKK-E12	Cholesterol	C14PEG2K	18:0 DDAB	30	30	2.5	37.5
LNP 20	cKK-E12	Cholesterol	C14PEG2K	18:0 DDAB	35	18	2.5	44.5
LNP 21	cKK-E12	Cholesterol	C14PEG2K	18:0 DDAB	45	42	2.5	10.5
LNP 22	cKK-E12	Cholesterol	C14PEG2K	18:0 DDAB	50	35	2.5	12.5
LNP 23	cKK-E12	Cholesterol	C14PEG2K	18:0 DDAB	52.5	15	2.5	30
LNP 24	cKK-E12	Cholesterol	C14PEG2K	18:0 DDAB	57.5	20	2.5	20
LNP 25	cKK-E12	Cholesterol	C14PEG2K	DOTAP	30	30	2.5	37.5
LNP 26	cKK-E12	Cholesterol	C14PEG2K	DOTAP	35	18	2.5	44.5
LNP 27	cKK-E12	Cholesterol	C14PEG2K	DOTAP	45	42	2.5	10.5
LNP 28	cKK-E12	Cholesterol	C14PEG2K	DOTAP	50	35	2.5	12.5
LNP 29	cKK-E12	Cholesterol	C14PEG2K	DOTAP	52.5	15	2.5	30
LNP 30	cKK-E12	Cholesterol	C14PEG2K	DOTAP	57.5	20	2.5	20

Screening 2

Name	Lipomer	Cholesterol	PEG	Helper Lipid	Lipomer	Cholesterol	PEG	Helper Lipid
LNP 31	cKK-E12	Cholesterol	C14PEG2K	DOPE	45	44	2	9
LNP 32	cKK-E12	Cholesterol	C14PEG2K	DOPE	35	46.5	2.5	16
LNP 33	cKK-E12	Cholesterol	C14PEG2K	DOPE	50	35	2.5	12.5
LNP 34	cKK-E12	Cholesterol	C14PEG2K	DOPE	30	30	1	39
LNP 35	cKK-E12	Cholesterol	C14PEG2K	DOPE	35	18	2.5	44.5
LNP 36	cKK-E12	Cholesterol	C14PEG2K	DOTAP	45	44	2	9
LNP 37	cKK-E12	Cholesterol	C14PEG2K	DOTAP	35	46.5	2.5	16
LNP 38	cKK-E12	Cholesterol	C14PEG2K	DOTAP	50	35	2.5	12.5
LNP 39	cKK-E12	Cholesterol	C14PEG2K	DOTAP	30	30	1	39
LNP 40	cKK-E12	Cholesterol	C14PEG2K	DOTAP	35	18	2.5	44.5
LNP 41	cKK-E12	Cholesterol	C14PEG2K	DOTMA	45	44	2	9
LNP 42	cKK-E12	Cholesterol	C14PEG2K	DOTMA	35	46.5	2.5	16
LNP 43	cKK-E12	Cholesterol	C14PEG2K	DOTMA	50	35	2.5	12.5
LNP 44	cKK-E12	Cholesterol	C14PEG2K	DOTMA	30	30	1	39
LNP 45	cKK-E12	Cholesterol	C14PEG2K	DOTMA	35	18	2.5	44.5
LNP 46	cKK-E12	20a-OH	C14PEG2K	DOPE	45	44	2	9
LNP 47	cKK-E12	20a-OH	C14PEG2K	DOPE	35	46.5	2.5	16
LNP 48	cKK-E12	20a-OH	C14PEG2K	DOPE	50	35	2.5	12.5
LNP 49	cKK-E12	20a-OH	C14PEG2K	DOPE	30	30	1	39
LNP 50	cKK-E12	20a-OH	C14PEG2K	DOPE	35	18	2.5	44.5
LNP 51	cKK-E12	20a-OH	C14PEG2K	DOTAP	45	44	2	9
LNP 52	cKK-E12	20a-OH	C14PEG2K	DOTAP	35	46.5	2.5	16
LNP 53	cKK-E12	20a-OH	C14PEG2K	DOTAP	50	35	2.5	12.5
LNP 54	cKK-E12	20a-OH	C14PEG2K	DOTAP	30	30	1	39
LNP 55	cKK-E12	20a-OH	C14PEG2K	DOTAP	35	18	2.5	44.5
LNP 56	cKK-E12	20a-OH	C14PEG2K	DOTMA	45	44	2	9
LNP 57	cKK-E12	20a-OH	C14PEG2K	DOTMA	35	46.5	2.5	16
LNP 58	cKK-E12	20a-OH	C14PEG2K	DOTMA	50	35	2.5	12.5
LNP 59	cKK-E12	20a-OH	C14PEG2K	DOTMA	30	30	1	39
LNP 60	cKK-E12	20a-OH	C14PEG2K	DOTMA	35	18	2.5	44.5
LNP 61	cKK-E12	20a-OH	C18PEG2K	DOPE	45	44	2	9
LNP 62	cKK-E12	20a-OH	C18PEG2K	DOPE	35	46.5	2.5	16
LNP 63	cKK-E12	20a-OH	C18PEG2K	DOPE	50	35	2.5	12.5
LNP 64	cKK-E12	20a-OH	C18PEG2K	DOPE	30	30	1	39
LNP 65	cKK-E12	20a-OH	C18PEG2K	DOPE	35	18	2.5	44.5
LNP 66	cKK-E12	20a-OH	C18PEG2K	DOTAP	45	44	2	9
LNP 67	cKK-E12	20a-OH	C18PEG2K	DOTAP	35	46.5	2.5	16
LNP 68	cKK-E12	20a-OH	C18PEG2K	DOTAP	50	35	2.5	12.5
LNP 69	cKK-E12	20a-OH	C18PEG2K	DOTAP	30	30	1	39
LNP 70	cKK-E12	20a-OH	C18PEG2K	DOTAP	35	18	2.5	44.5
LNP 71	cKK-E12	20a-OH	C18PEG2K	DOTMA	45	44	2	9
LNP 72	cKK-E12	20a-OH	C18PEG2K	DOTMA	35	46.5	2.5	16
LNP 73	cKK-E12	20a-OH	C18PEG2K	DOTMA	50	35	2.5	12.5
LNP 74	cKK-E12	20a-OH	C18PEG2K	DOTMA	30	30	1	39
LNP 75	cKK-E12	20a-OH	C18PEG2K	DOTMA	35	18	2.5	44.5
LNP 76	cKK-E12	Cholesterol	C18PEG2K	DOPE	45	44	2	9
LNP 77	cKK-E12	Cholesterol	C18PEG2K	DOPE	35	46.5	2.5	16
LNP 78	cKK-E12	Cholesterol	C18PEG2K	DOPE	50	35	2.5	12.5
LNP 79	cKK-E12	Cholesterol	C18PEG2K	DOPE	30	30	1	39
LNP 80	cKK-E12	Cholesterol	C18PEG2K	DOPE	35	18	2.5	44.5
LNP 81	cKK-E12	Cholesterol	C18PEG2K	DOTAP	45	44	2	9
LNP 82	cKK-E12	Cholesterol	C18PEG2K	DOTAP	35	46.5	2.5	16
LNP 83	cKK-E12	Cholesterol	C18PEG2K	DOTAP	50	35	2.5	12.5
LNP 84	cKK-E12	Cholesterol	C18PEG2K	DOTAP	30	30	1	39
LNP 85	cKK-E12	Cholesterol	C18PEG2K	DOTAP	35	18	2.5	44.5
LNP 86	cKK-E12	Cholesterol	C18PEG2K	DOTMA	45	44	2	9
LNP 87	cKK-E12	Cholesterol	C18PEG2K	DOTMA	35	46.5	2.5	16
LNP 88	cKK-E12	Cholesterol	C18PEG2K	DOTMA	50	35	2.5	12.5
LNP 89	cKK-E12	Cholesterol	C18PEG2K	DOTMA	30	30	1	39
LNP 90	cKK-E12	Cholesterol	C18PEG2K	DOTMA	35	18	2.5	44.5

Screening 3

Name	Lipomer	Cholesterol	PEG	Helper Lipid	Lipomer	Cholesterol	PEG	Helper Lipid
LNP 91	KB11	Cholesterol	C14PEG	DOTAP	45.0	38.8	2.5	13.7
LNP 92	KB11	Cholesterol	C14PEG	DOTAP	50.0	35.0	2.5	12.5
LNP 93	KB11	20a-OH	C14PEG	DOTAP	40.0	42.7	2.5	14.8
LNP 94	KB11	20a-OH	C14PEG	DOTAP	45.0	38.8	2.5	13.7
LNP 95	KB11	20a-OH	C14PEG	DOTAP	45.0	41.5	1.5	12.0
LNP 96	KB12	Cholesterol	C14PEG	DOTAP	35.0	46.5	2.5	16.0
LNP 97	KB12	Cholesterol	C14PEG	DOTAP	35.0	43.5	1.5	20.0
LNP 98	KB11	Cholesterol	C14PEG	DOPE	45.0	38.8	2.5	13.7
LNP 99	KB11	Cholesterol	C14PEG	DOPE	50.0	35.0	2.5	12.5
LNP 100	KB11	20a-OH	C14PEG	DOPE	40.0	42.7	2.5	14.8
LNP 101	KB11	20a-OH	C14PEG	DOPE	45.0	38.8	2.5	13.7
LNP 102	KB11	20a-OH	C14PEG	DOPE	45.0	41.5	1.5	12.0
LNP 103	KB12	Cholesterol	C14PEG	DOPE	35.0	46.5	2.5	16.0
LNP 104	KB12	Cholesterol	C14PEG	DOPE	35.0	43.5	1.5	20.0
LNP 105	KB12	20a-OH	C14PEG	DOPE	35.0	46.5	2.5	16.0
LNP 106	KB12	20a-OH	C14PEG	DOPE	45.0	38.8	2.5	13.7
LNP 107	KB12	20a-OH	C14PEG	DOPE	50.0	35.0	2.5	12.5
LNP 108	KB12	20a-OH	C14PEG	DOPE	35.0	43.5	1.5	20.0
LNP 109	KB12	20a-OH	C14PEG	DOPE	40.0	42.5	1.5	16.0
LNP 110	KB12	20a-OH	C14PEG	DOTAP	35.0	46.5	2.5	16.0
LNP 111	KB12	20a-OH	C14PEG	DOTAP	45.0	38.8	2.5	13.7
LNP 112	KB12	20a-OH	C14PEG	DOTAP	50.0	35.0	2.5	12.5
LNP 113	KB12	20a-OH	C14PEG	DOTAP	35.0	43.5	1.5	20.0
LNP 114	KB12	20a-OH	C14PEG	DOTAP	40.0	42.5	1.5	16.0
LNP 115	KB15	Cholesterol	C14PEG	DOPE	35.0	46.5	2.5	16.0
LNP 116	KB15	Cholesterol	C14PEG	DOPE	40.0	42.7	2.5	14.8
LNP 117	KB15	Cholesterol	C14PEG	DOPE	45.0	38.8	2.5	13.7
LNP 118	KB15	Cholesterol	C14PEG	DOPE	50.0	35.0	2.5	12.5
LNP 119	KB15	Cholesterol	C14PEG	DOPE	40.0	42.5	1.5	16.0
LNP 120	KB15	20a-OH	C14PEG	DOPE	35.0	46.5	2.5	16.0
LNP 121	KB15	20a-OH	C14PEG	DOPE	40.0	42.7	2.5	14.8
LNP 122	KB15	20a-OH	C14PEG	DOPE	45.0	38.8	2.5	13.7
LNP 123	KB15	20a-OH	C14PEG	DOPE	35.0	43.5	1.5	20.0
LNP 124	KB15	Cholesterol	C14PEG	DOTAP	35.0	46.5	2.5	16.0
LNP 125	KB15	Cholesterol	C14PEG	DOTAP	40.0	42.7	2.5	14.8
LNP 126	KB15	Cholesterol	C14PEG	DOTAP	45.0	38.8	2.5	13.7
LNP 127	KB15	Cholesterol	C14PEG	DOTAP	50.0	35.0	2.5	12.5
LNP 128	KB15	Cholesterol	C14PEG	DOTAP	40.0	42.5	1.5	16.0
LNP 129	KB15	20a-OH	C14PEG	DOTAP	35.0	46.5	2.5	16.0
LNP 130	KB15	20a-OH	C14PEG	DOTAP	40.0	42.7	2.5	14.8
LNP 131	KB15	20a-OH	C14PEG	DOTAP	45.0	38.8	2.5	13.7
LNP 132	KB16	Cholesterol	C14PEG	DOPE	40.0	42.7	2.5	14.8
LNP 133	KB16	Cholesterol	C14PEG	DOPE	40.0	42.5	1.5	16.0
LNP 134	KB16	20a-OH	C14PEG	DOPE	35.0	46.5	2.5	16.0
LNP 135	KB16	20a-OH	C14PEG	DOPE	40.0	42.7	2.5	14.8
LNP 136	KB16	20a-OH	C14PEG	DOPE	45.0	38.8	2.5	13.7
LNP 137	KB16	20a-OH	C14PEG	DOPE	50.0	35.0	2.5	12.5
LNP 138	KB16	20a-OH	C14PEG	DOPE	45.0	41.5	1.5	12.0
LNP 139	KB16	Cholesterol	C14PEG	DOTAP	40.0	42.7	2.5	14.8
LNP 140	KB16	Cholesterol	C14PEG	DOTAP	40.0	42.5	1.5	16.0
LNP 141	KB15	20a-OH	C14PEG	DOTAP	35.0	43.5	1.5	20.0
LNP 142	KB16	20a-OH	C14PEG	DOTAP	35.0	46.5	2.5	16.0
LNP 143	KB16	20a-OH	C14PEG	DOTAP	40.0	42.7	2.5	14.8
LNP 144	KB16	20a-OH	C14PEG	DOTAP	45.0	38.8	2.5	13.7
LNP 145	KB16	20a-OH	C14PEG	DOTAP	50.0	35.0	2.5	12.5
LNP 146	KB16	20a-OH	C14PEG	DOTAP	45.0	41.5	1.5	12.0
TAACTCGG								
TGCCTTGA								
CCAGAGTA								

Fig. S1. Detailed outline of total 150 LNPs with varying composition for screenings

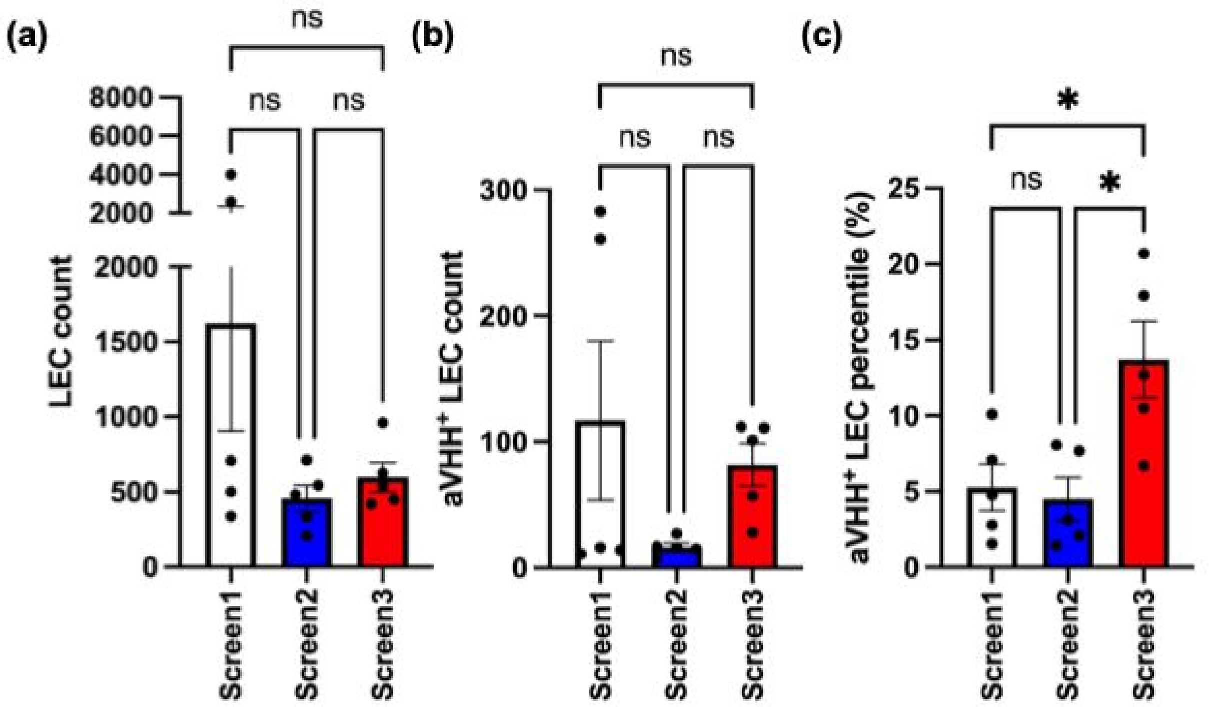

(d)

Screen	LEC count	aVHH⁺ LEC count	aVHH⁺ LEC %
Screen 1	503	14	2.78
	709	11	1.55
	338	16	4.73
	2577	261	10.1
	3984	283	7.1
Screen 2	484	7	1.45
	544	17	3.12
	208	16	7.69
	334	27	8.08
	713	15	2.1
Screen 3	450	57	12.7
	419	28	6.68
	962	101	10.5
	535	111	20.7
	624	112	17.9

Fig. S2. LEC and LEC/aVHH^{+} count for screens. LEC count at screens for SANDS **(a)**.

LEC/ aVHH + count at screens for SANDS **(b)** aVHH +/LEC percentile **(c)** Their table **(d).**

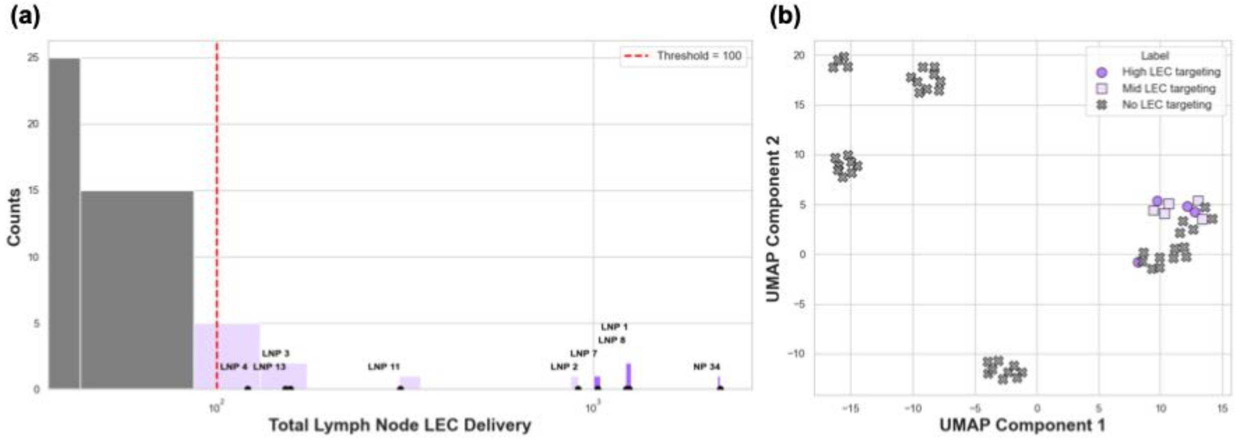

Fig. S3. SANDS library visualization via histogram and UMAP for the selection of leading LNP candidates. (a) Histogram for the selection of leading LNP candidates **(b)**. Visualization of the cluster via Uniform Manifold Approximation and Projection (UMAP).

(a)

LNP	Diameter	PDI	Zeta potential (mV)	EE%
LNP1	87.0	0.2	26.2	79.6
LNP2	94.0	0.4	24.3	75.5
LNP3	77.4	0.3	9.04	87.9
LNP4	57.1	0.2	-2.22	93.7
LNP7	71.6	0.3	8.95	76.1
LNP11	76.8	0.4	14.0	71.1
LNP13	143.8	0.2	2.91	25.3
LNP21	90.1	0.3	16.88	74.2
LNP25	145.1	0.4	11.86	68.8
LNP27	90.5	0.2	13.48	59.8

(b)

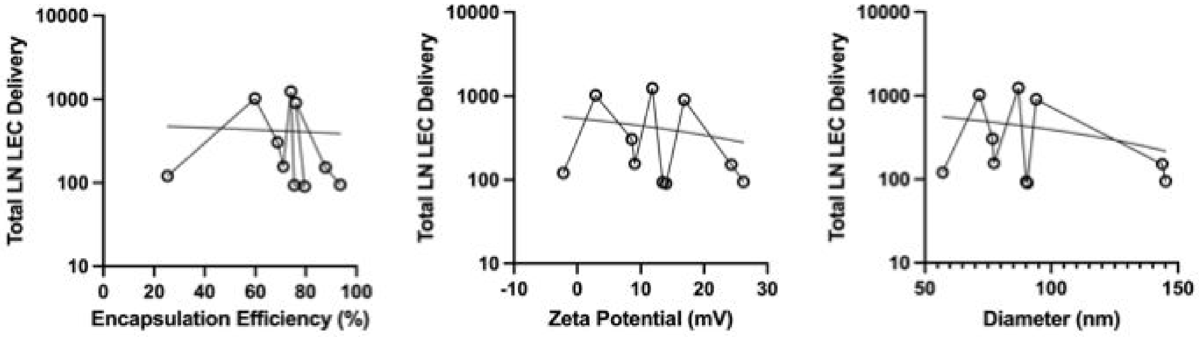

(c)

LNP Parameters	Diameter	Zeta potential (mV)	EE%
p-value	0.4874	0.5926	0.8862
R^2	0.06212	0.03737	0.00272

Fig. S4. LNP parameters did not show a linear relationship to lymphatic node delivery. **(a)**. Leading LNP candidates and non-LEC targeting LNPs were measured at diameter (nm), Zeta potential (mV), and encapsulation efficiency (EE%). Linear regression to determine the linearity or correlation between LNP parameters and LN delivery. Its plot **(b)** and results **(c)** were shown above. None of the LNP parameters showed significant linearity to LN delivery.

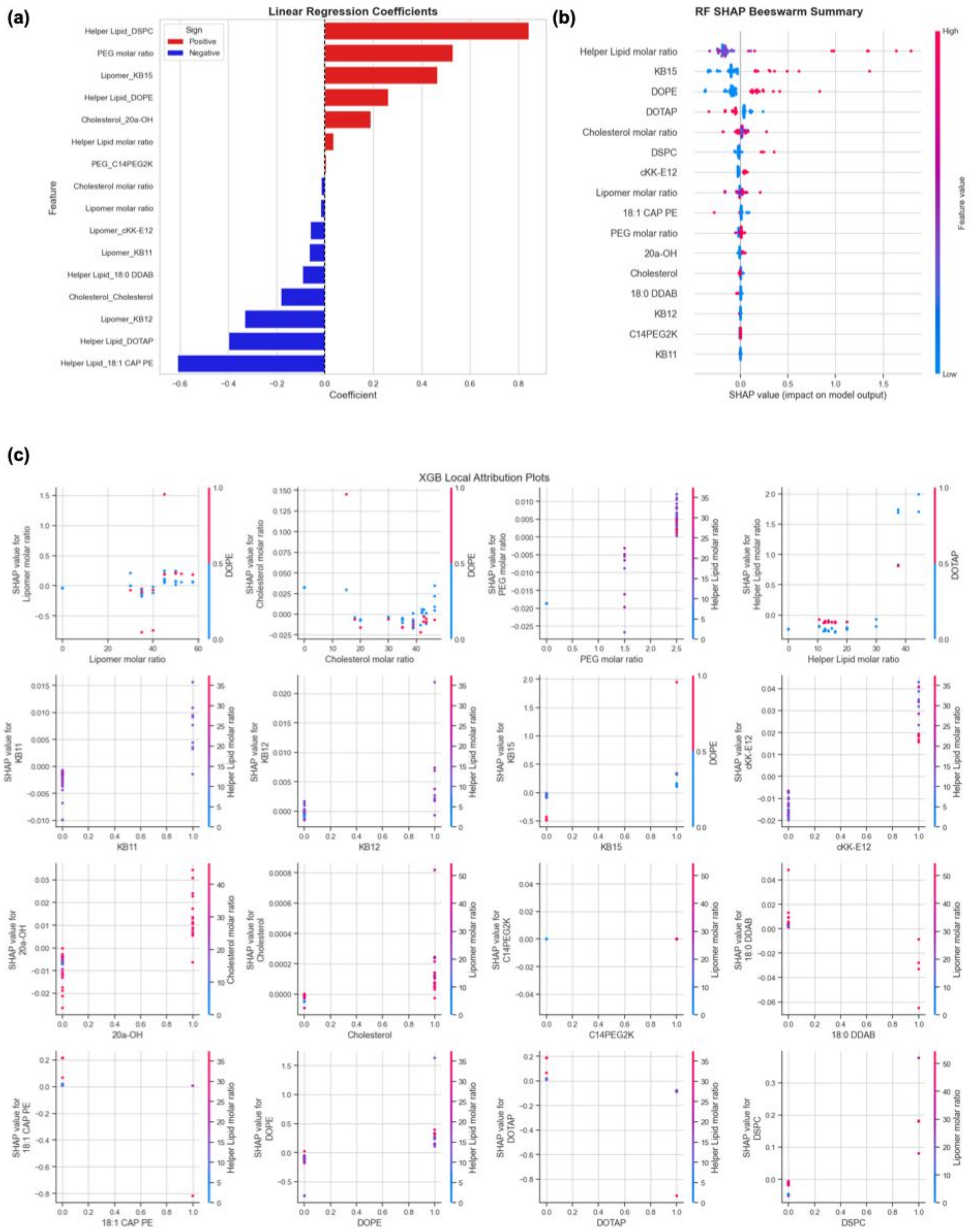
(a)
Linear Regression Coefficients
Sign
Positive
Negative
Helper Lipid_DSPC
PEG molar ratio
Lipomer_KB15
Helper Lipid_DOPE
Cholesterol_20a-OH
Helper Lipid molar ratio
PEG_C14PEG2K
Cholesterol molar ratio
Lipomer molar ratio
Lipomer_cKK-E12
Lipomer_KB11
Helper Lipid_18:0 DDAB
Cholesterol_Cholesterol
Lipomer_KB12
Helper Lipid_DOTAP
Helper Lipid_18:1 CAP PE
Feature
Coefficient
(b)
RF SHAP Beeswarm Summary
Helper Lipid molar ratio
KB15
DOPE
DOTAP
Cholesterol molar ratio
DSPC
cKK-E12
Lipomer molar ratio
18:1 CAP PE
PEG molar ratio
20a-OH
Cholesterol
18:0 DDAB
KB12
C14PEG2K
KB11
SHAP value (impact on model output)
High
Low
Feature value
(c)
XGB Local Attribution Plots
SHAP value for Lipomer molar ratio
Lipomer molar ratio
SHAP value for Cholesterol molar ratio
Cholesterol molar ratio
SHAP value for PEG molar ratio
PEG molar ratio
SHAP value for Helper Lipid molar ratio
Helper Lipid molar ratio
SHAP value for KB11
KB11
SHAP value for KB12
KB12
SHAP value for KB15
KB15
SHAP value for cKK-E12
cKK-E12
SHAP value for 20a-OH
20a-OH
SHAP value for Cholesterol
Cholesterol
SHAP value for C14PEG2K
C14PEG2K
SHAP value for 18:0 DDAB
18:0 DDAB
SHAP value for 18:1 CAP PE
18:1 CAP PE
SHAP value for DOPE
DOPE
SHAP value for DOTAP
DOTAP
SHAP value for DSPC
DSPC
Cholesterol molar ratio
Lipomer molar ratio

Fig. S5. Coefficient plot of linear regression (a), feature importance plot of random forest (RF) (b), and local attribution plot of Xgboost (XGB) (c).

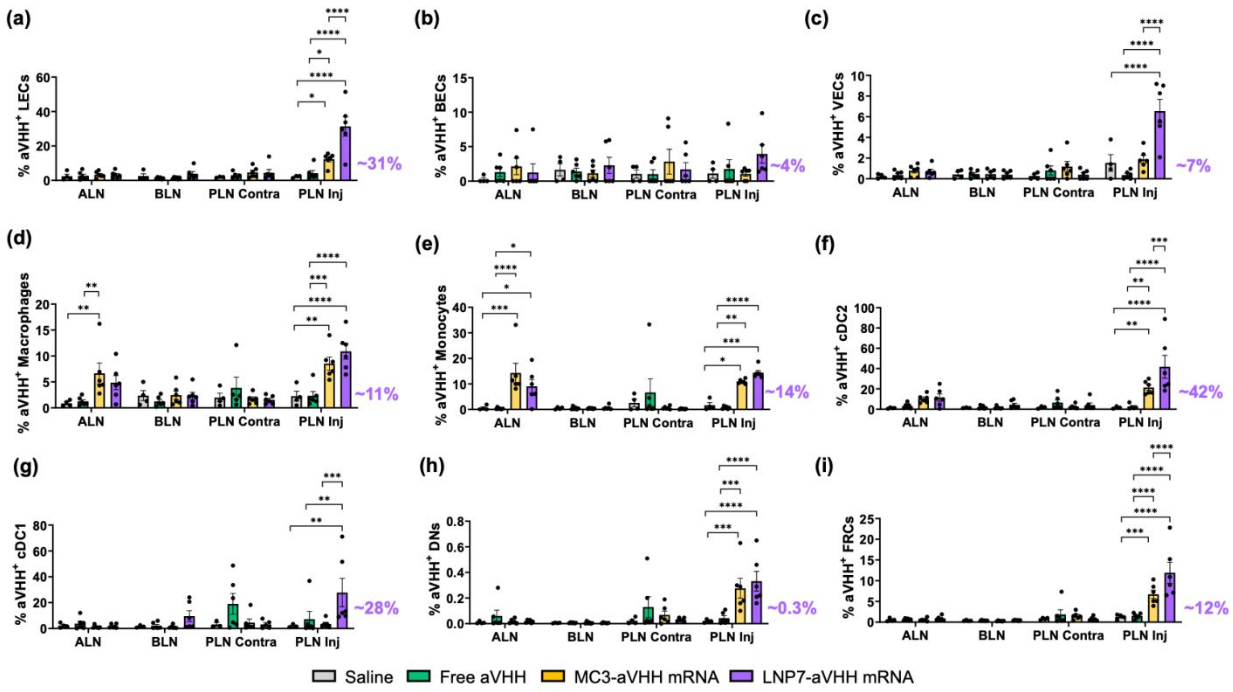

Fig. S6. Lymphatic-specific uptake of LNP7. Percentage of aVHH$^+$: (a) LECs, **(b)** BECs, **(c)** VECs, **(d)** Macrophages, **(e)** Monocytes, **(f)** cDC2, **(g)** cDC1, **(h)** DNs, and **(i)** FRCs from ALN, BLN, PLN Contra (non-injected), and PLN Inj that have successfully taken up saline (gray), free aVHH (green), MC3 (gold) and LNP7 (purple). Each data point corresponds to an independent experiment (ALN: $N_{Saline} = 4$, $N_{Free\ aVHH} = 6$, $N_{MC3} = 6$, and $N_{LNP7} = 6$; BLN: $N_{Saline} = 4$, $N_{Free\ aVHH} = 6$, $N_{MC3} = 6$, and $N_{LNP7} = 6$, PLN Contra: $N_{Saline} = 4$, $N_{Free\ aVHH} = 6$, $N_{MC3} = 6$, and $N_{LNP7} = 6$; PLN Inj: $N_{Saline} = 4$, $N_{Free\ aVHH} = 6$, $N_{MC3} = 6$, and $N_{LNP7} = 6$), and error bars represent the standard error of the mean. Solid lines above plots indicate a pairwise comparison for significance using a two-way ANOVA with Tukey's multiple comparisons test with $p < 0.05$ (*), $p < 0.01$ (**), $p <$

0.001 (***), and $p < 0.0001$ (****). Note: *y*-axes are scaled independently for each cell type to allow clear visualization of relative differences within each population.

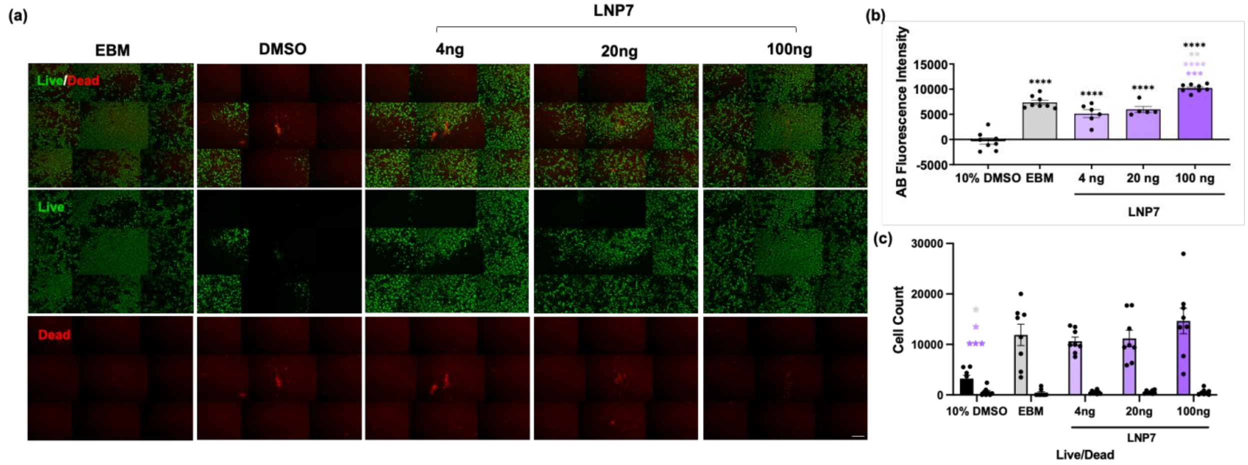

Fig. S7. LNP7 does not significantly lower the viability of LECs. (a) Live (Calcein-AM, green) and Dead (TOTO-3, red) staining of human LEC monolayers after treatment with controls or LNP7. (Scale bar = 200 µm.) Contrast was enhanced post-acquisition equally in all image panels for ease of viewing. Quantification of **(b)** Alamar Blue fluorescence intensity (metabolic activity) and **(c)** Live/Dead staining. **(b)** Color-coordinated asterisks indicate comparison with the corresponding treatment using one-way ANOVA with Tukey's multiple comparisons test and robust regression and outlier removal (ROUT) method to identify and remove outliers with $p < 0.01$ (**), $p < 0.001$ (***), and $p < 0.0001$ (****). **(c)** Cell count of Live (left bar) and Dead (right bar) positive stain. Asterisks indicate significant differences by Sídák's multiple comparisons test with $p < 0.05$ (*), $p < 0.01$ (**), $p < 0.001$ (***), and $p < 0.0001$ (****).

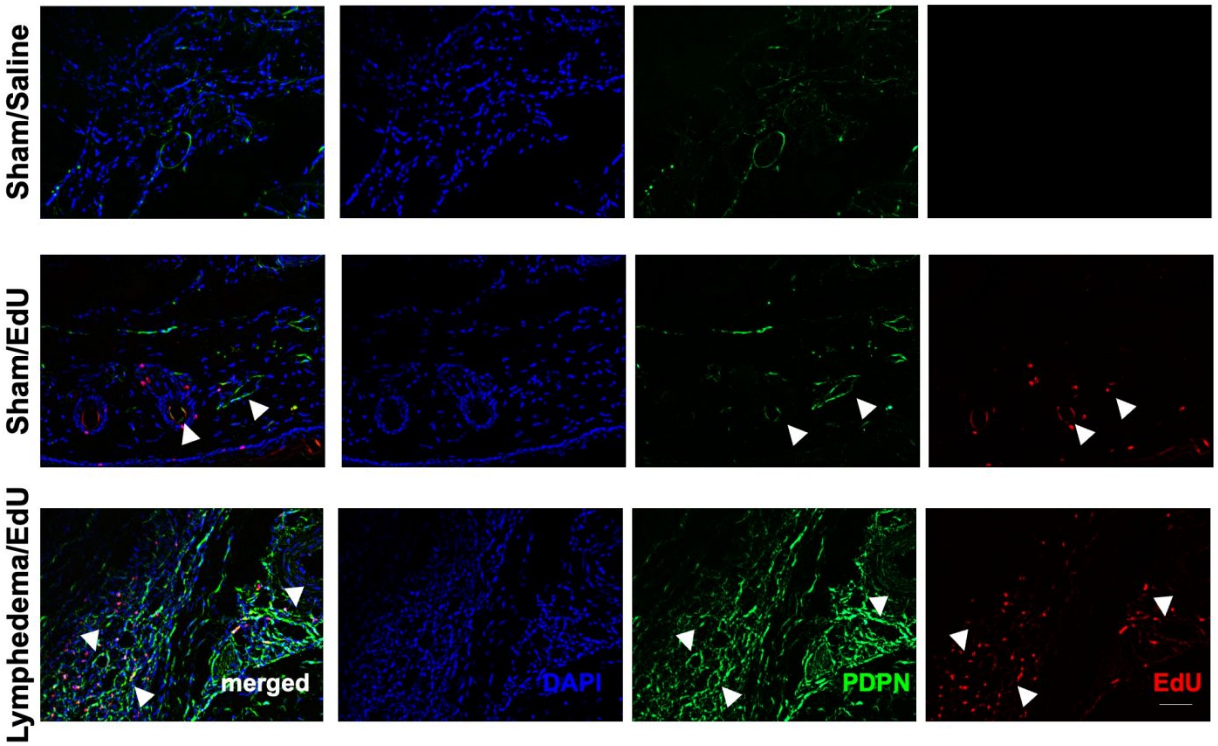

Fig. S8. Immunofluorescence controls in tail sections. Immunofluorescence micrographs of tail LV segments for sham mice injected with saline (negative control), sham mice injected with EdU, and lymphedema mice injected with EdU 7 days post-surgery for merged, DAPI (blue), PDPN (green), and EdU (red). (20x objective; Scale bar = 50 µm.) Arrows indicate EdU and PDPN double positive LEC. The contrast was enhanced post-acquisition equally in all image panels for ease of viewing.

	Characterization (N=4)			
	**mRNA Cargo**	**aVHH**	**Cy3-miRNA**	**VEGFC**
MC3	Diameter (nm)	76.43 ± 11.70	71.91 ± 9.67	97.23 ± 25.13
	PDI	0.15 ± 0.13	0.13 ± 0.05	0.33 ± 0.11
	Encapsulation Efficiency (%)	88.97 ± 3.31	90.53 ± 1.01	71.09 ± 19.57
	Total mRNA Concentration (μg/mL)	117.10 ± 6.77	233.87 ± 28.89	154.90 ± 6.505
	Encapsulated mRNA Concentration (μg/mL)	104.15 ± 0.22	211.7 ± 0.29	110.10 ± 1.27
LNP7	Diameter (nm)	71.68 ± 11.88	79.03 ± 19.67	83.37± 16.74
	PDI	0.27 ± 0.06	0.22 ± 0.13	0.32 ± 0.10
	Encapsulation Efficiency (%)	76.13 ± 4.55	89.97 ± 1.57	55.22 ± 4.65
	Total mRNA Concentration (μg/mL)	77.96 ± 8.03	149.9 ± 18.61	81.37 ± 11.82
	Encapsulated mRNA Concentration (μg/mL)	59.36 ± 0.37	134.86 ± 0.29	44.93 ± 0.55

Fig. S9. Characterization of MC3 and LNP7 loaded with different nucleic acid cargos, namely aVHH mRNA, miRNA-cy3, and VEGFC mRNA (n=4 for each). Hydrodynamic diameter (nm), PDI, encapsulation efficiency (%), total mRNA concentration (mg/mL), encapsulated mRNA concentration (mg/mL) of MC3 and LNP7 loaded with aVHH mRNA and VEGFC mRNA.

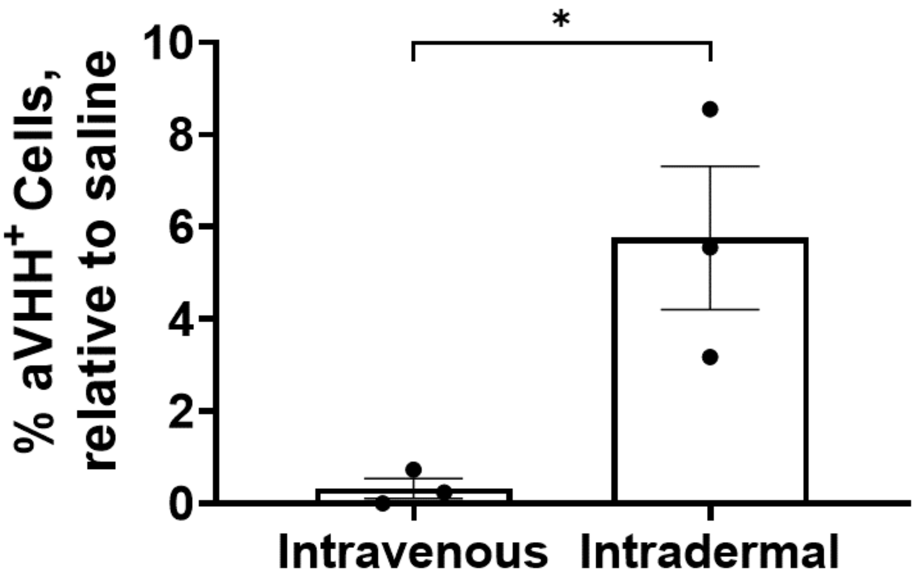

Fig. S10. Effect of the route of administration on LNP delivery. Percentage of $aVHH^+$ cells (after gating for Live/Dead, $CD31^+/PDPN^+$, and relative to saline (meaning, the saline measurement was subtracted from the corresponding uptake)) in PLN that have successfully taken up the LNP cargo after intravenous and intradermal injections during the LNP screening study. Each data point corresponds to an independent experiment ($N_{Intravenous}$ = 3 and $N_{Intradermal}$ = 3), and error bars represent the standard error of the mean. The solid line above plots indicates a pairwise comparison for significance using an unpaired *t*-test with $p < 0.05$ (*).

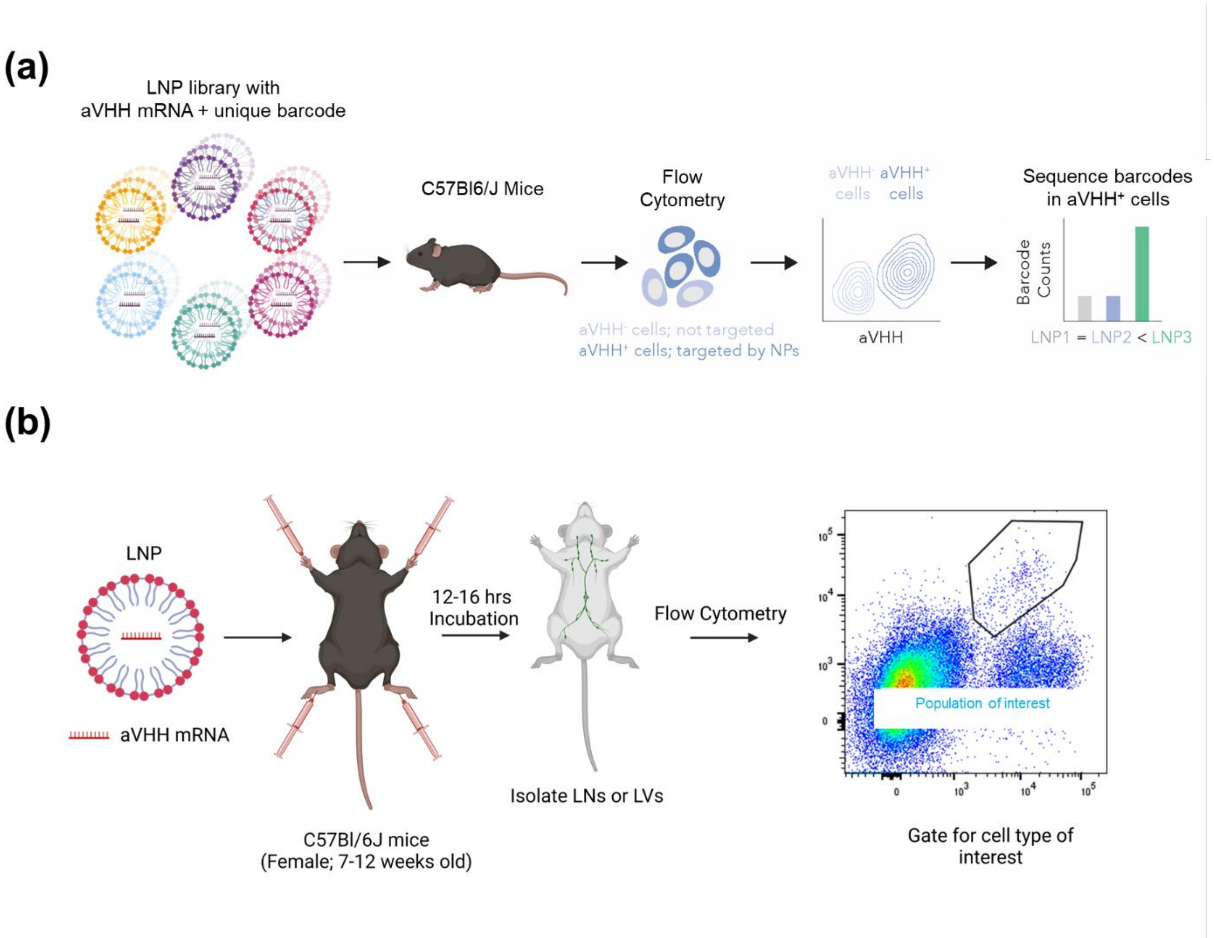

Fig. S11. Engineer LEC-targeting LNPs. (a) LNP libraries with aVHH mRNA and unique DNA barcode were administered to C57Bl6/J mice. aVHH$^+$ cells were isolated by FACS and DNA barcodes were sequenced. **(b)** LNPs were intradermally injected in C57Bl/6J mice and 12-16 hrs later the downstream LNs and LVs were collected for flow cytometry. Control animals are ID injected with appropriate volumes of saline.

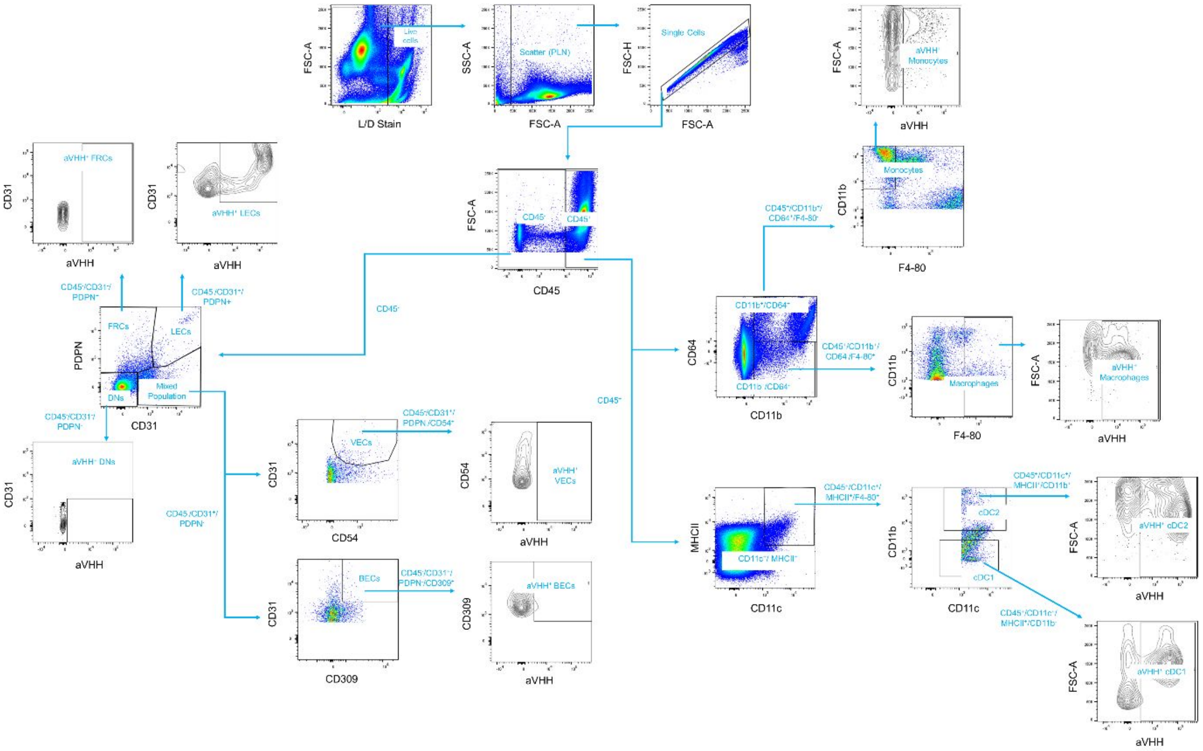

Fig. S12. Representative gating strategies for FACS for various cell populations present in LNs. Identifying the following cell populations via appropriate gating as seen above (after Live/Dead gating); LECs: $CD45^-/CD31^+/PDPN^+$, VECs: $CD45^-/CD31^+/PDPN^-/CD54^+$, BECs: $CD45^-/CD31^+/PDPN^-/CD309^+$, FRCs: $CD45^-/CD31^-/PDPN^+$, DNs: $CD45^-/CD31^-/PDPN^-$, Monocytes: $CD45^+/CD11b^+/CD64^+/F4\text{-}80^-$, Macrophages: $CD45^+/CD11b^+/CD64^-/F4\text{-}80^+$, cDC2: $CD45^+/CD11c^+/MHCII^+/CD11b^+$, and cDC1: $CD45^+/CD11c^+/MHCII^+/CD11b^-$.

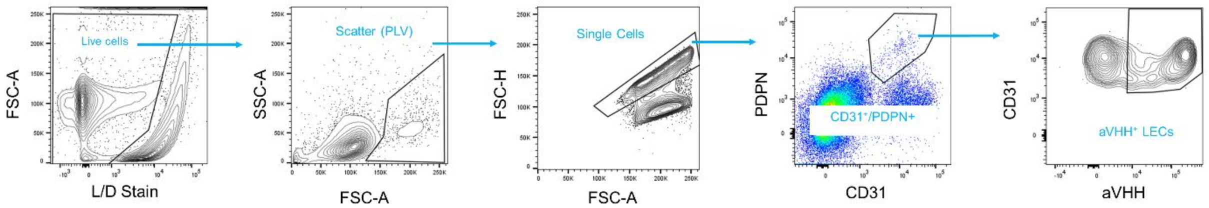

Fig. S13. Representative gating strategies for FACS for LECs in LVs. Identifying the LEC population via appropriate gating (after Live/Dead gating) as seen above; LECs: $CD31^+$/$PDPN^+$.

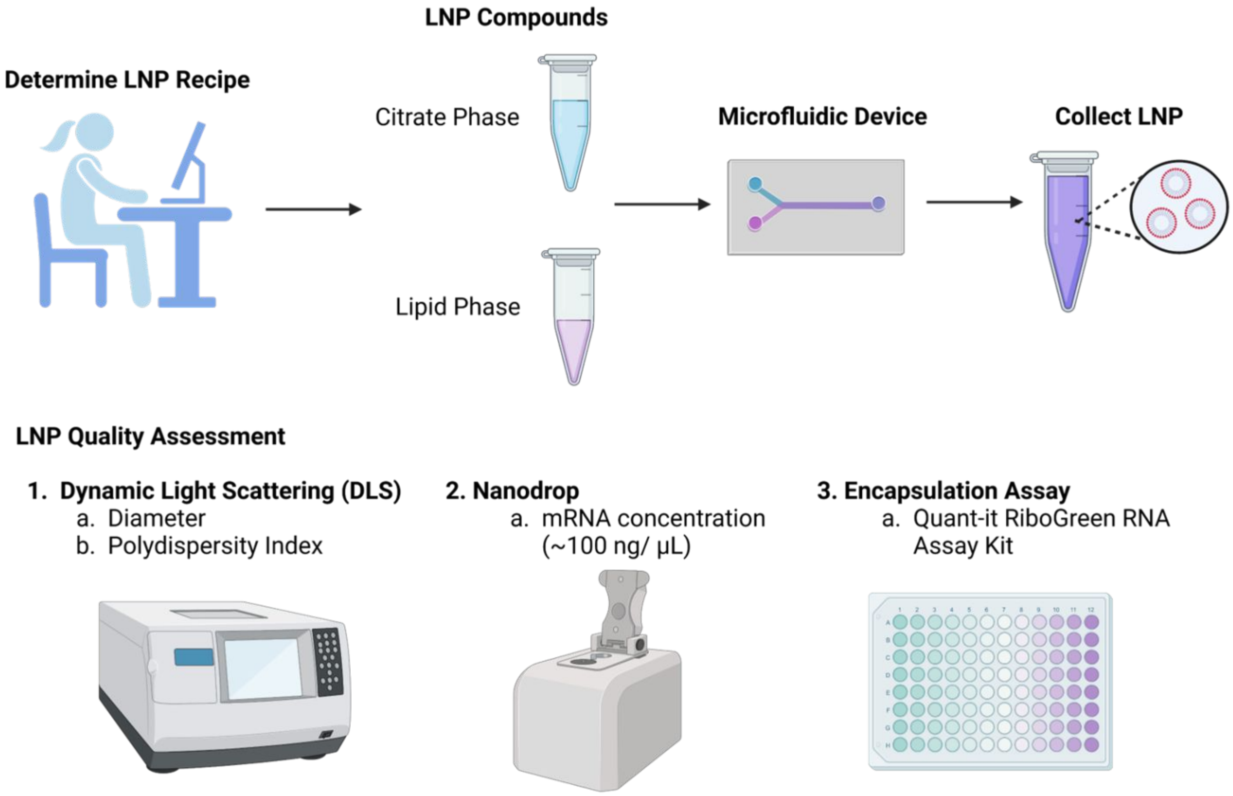

Fig. S14. Steps for LNP formulation and quality assessment. The LNP recipe was determined, and the corresponding citrate and lipid phases were combined in the microfluidic device for LNP formulation. The quality of the formulated LNP was evaluated using dynamic light scattering (DLS) to measure the diameter and polydispersity index (PDI), nanodrop to measure the mRNA concentration, and encapsulation assay to quantify the corresponding encapsulation efficiency.

Supplementary tables

LNP#	Lipomer	Cholesterol	PEG	Helper lipid	Lipomer mole %	Cholesterol mole %	PEG mole %	Helper lipid mole %
LNP1	cKK-E12	Cholesterol	C14PEG2K	DSPC	30	30	2.5	37.5
LNP2	cKK-E12	Cholesterol	C14PEG2K	DSPC	35	18	2.5	44.5
LNP3	cKK-E12	Cholesterol	C14PEG2K	DSPC	45	42	2.5	10.5
LNP4	cKK-E12	Cholesterol	C14PEG2K	DSPC	50	35	2.5	12.5
LNP7	cKK-E12	Cholesterol	C14PEG2K	DOPE	30	30	2.5	37.5
LNP11	cKK-E12	Cholesterol	C14PEG2K	DOPE	52.5	15	2.5	30
MC3	MC3	Cholesterol	C14PEG2K	DSPC	50	38.5	1.5	10

Table S1. Compounds and molar ratios included in lead LEC-specific LNP candidates, namely LNP1, LNP2, LNP3, LNP4, LNP7, LNP11, and MC3. Unique LNPs were formulated with different combinations of compounds and molar ratios of ionizable lipid, cholesterol, PEG, and helper lipid.

Type of LN	Administration	LEC Count	aVHH$^+$ Cell Count
ALN	Saline	2182	37
	LNP1	1022	543
	LNP2	1467	697
	LNP3	948	134
	LNP4	997	134
	LNP7	2757	977
	LNP11	372	37
BLN	Saline	2016	39
	LNP1	2139	376
	LNP2	2169	715
	LNP3	1971	572
	LNP4	3319	159
	LNP7	2204	743
	LNP11	3631	239
PLN	Saline	95	2
	LNP1	388	139
	LNP2	105	31
	LNP3	771	144
	LNP4	118	49
	LNP7	782	292
	LNP11	230	52

Table S2. Average LEC count and LEC/aVHH$^+$ cell count for lead LEC-specific LNP candidates, namely LNP1, LNP2, LNP3, LNP4, LNP7, and LNP11.

		Cell Type Count								
**Type of LN**	**Administration**	**Macrophages**	**Monocytes**	**cDC2**	**cDC1**	**DNs**	**FRCs**	**LECs**	**BECs**	**VECs**
**ALN**	**Saline**	4036	1294	2914	1008	114907	6175	7099	237	1269
	**Free aVHH**	4970	1606	4155	1356	270150	6045	10462	744	3796
	**MC3**	4013	67256	4264	2682	155750	7848	7993	145	1995
	**LNP7**	4665	10492	6018	3885	337655	8294	7802	167	1879
**BLN**	**Saline**	7113	2666	8368	2474	105104	13394	10007	296	2267
	**Free aVHH**	10295	4097	11666	3698	168662	15316	16065	574	6351
	**MC3**	8562	6471	12017	4630	224232	21738	15810	249	5418
	**LNP7**	7940	4129	12104	3390	227405	14637	10541	310	3433
**PLN Contralateral**	**Saline**	1790	728	1417	541	18325	2056	3034	3475	488
	**Free aVHH**	1069	439	915	636	15212	1273	3686	45	624
	**MC3**	1302	651	1486	719	13422	2742	1267	28	381
	**LNP7**	1784	927	1934	912	29328	2412	4061	62	1111
**PLN Injected**	**Saline**	2054	599	1330.25	336	28092	1791	3272	56.25	632
	**Free aVHH**	4425	1737	1680	874	27735	1990	4153	70.5	1002
	**MC3**	7739	25134	2737	2445	34778	4618	4220	72	1091
	**LNP7**	7568	25225	3605	2586	69388	5089	5042	85	1058
**PLV**	**Saline**	NA	NA	NA	NA	NA	NA	185	NA	NA
	**Free aVHH**	NA	NA	NA	NA	NA	NA	188	NA	NA
	**MC3**	NA	NA	NA	NA	NA	NA	189	NA	NA
	**LNP7**	NA	NA	NA	NA	NA	NA	269	NA	NA

Table S3. Average cell count for the various cell types found in ALN, BLN, PLN Contralateral (non-injected), PLN injected, and PLV for saline-, free aVHH-, MC3-, and LNP7-injected animals.

		aVHH⁺ Cell Count								
**Type of LN**	**Administration**	**Macrophages**	**Monocytes**	**cDC2**	**cDC1**	**DNs**	**FRCs**	**LECs**	**BECs**	**VECs**
**ALN**	**Saline**	43	3	31	21	8.5	35	89	1	3
	**Free aVHH**	45	3	106	37	52	38	406	3	15
	**MC3**	320	876	437	31	31	44	263	1	18
	**LNP7**	201	1142	781	82	55	62	192	1	14
**BLN**	**Saline**	160	5	492	246	6	49	156	5	13
	**Free aVHH**	150	10	170	75	10	67	226	115	44
	**MC3**	287	20	162	47	14	65	254	3	41
	**LNP7**	152	28	578	441	14	57	409	7	21
**PLN Contralateral**	**Saline**	50	2.5	37	52	3	17	65	0.5	2
	**Free aVHH**	37	2	97	149	5	9	158	1	12
	**MC3**	25	4	43	35	12	68	45	1	4
	**LNP7**	28	2	82	75	8	16	89	2	8
**PLN Injected**	**Saline**	73	2	31	13	6	21	98	1	7
	**Free aVHH**	78	11	29	11	10	21	79	1	8
	**MC3**	772	2755	521	86	78	296	591	1	20
	**LNP7**	888	3681	1677	978	202	568	1690	3	77
**PLV**	**Saline**	NA	NA	NA	NA	NA	NA	3	NA	NA
	**Free aVHH**	NA	NA	NA	NA	NA	NA	3	NA	NA
	**MC3**	NA	NA	NA	NA	NA	NA	18	NA	NA
	**LNP7**	NA	NA	NA	NA	NA	NA	104	NA	NA

Table S4. Average aVHH⁺ cell count for the various cell types found in ALN, BLN, PLN Contralateral (non-injected), PLN injected, and PLV for saline-, free aVHH, MC3-, and LNP7-injected animals.
